# Investigating the *AC079305/DUSP1* Axis as Oxidative Stress-Related Signatures and Immune Infiltration Characteristics in Ischemic Stroke

**DOI:** 10.1155/2022/8432352

**Published:** 2022-06-14

**Authors:** Jiaxin Fan, Shuai Cao, Mengying Chen, Qingling Yao, Xiaodong Zhang, Shuang Du, Huiyang Qu, Yuxuan Cheng, Shuyin Ma, Meijuan Zhang, Yizhou Huang, Nan Zhang, Kaili Shi, Shuqin Zhan

**Affiliations:** ^1^Department of Neurology, The Second Affiliated Hospital of Xi'an Jiaotong University, Xi'an, China; ^2^Department of Orthopedics, The Second Affiliated Hospital of Xi'an Jiaotong University, Xi'an, China

## Abstract

**Background:**

Oxidative stress (OS) and immune inflammation play complex intersections in the pathophysiology of ischemic stroke (IS). However, a competing endogenous RNA- (ceRNA-) based mechanism linked to the intersections in IS has not been explored. We aimed to identify potential OS-related signatures and analyze immune infiltration characteristics in IS.

**Methods:**

Three datasets (GSE22255, GSE110993, and GSE140275) from the GEO database were extracted. Differentially expressed long noncoding RNAs, microRNAs, and messenger RNAs (DElncRNAs, DEmiRNAs, and DEmRNAs) between IS patients and controls were identified. Gene Ontology (GO) and Kyoto Encyclopedia of Genes and Genomes (KEGG) enrichment analysis were explored. Moreover, a triple ceRNA network was constructed to reveal transcriptional regulation mechanisms. A comprehensive strategy among least absolute shrinkage and selection operator (LASSO) regression, DEmRNAs, uprelated DEmRNAs, and OS-related genes was adopted to select the best signature. Then, we evaluated and verified the discriminant ability of the signature via receiver operating characteristic (ROC) analysis. Immune infiltration characteristics were explored via the CIBERSORT algorithm. Moreover, the best signature was verified via qPCR and western blot methods in rat brain tissues and PC12 cells.

**Results:**

11 DEmRNAs were identified totally. Enrichment analysis showed that the DEmRNAs were primarily concentrated in MAPK-associated biological processes and immune or inflammation-involved pathways. *DUSP1* was identified as the best signature with an area under the ROC curve of 73.5% (95%CI = 57.02-89.98, sensitivity = 95%, and specificity = 60%) in GSE22255 and 100.0% (95%CI = 100.00-100.00, sensitivity = 100%, and specificity = 100%) in GSE140275. Importantly, we also identified the *AC079305/DUSP1* axis in the ceRNA network. Immune infiltration showed that resting mast cells infiltrate less in IS patients compared with controls. And *DUSP1* was negatively correlated with resting mast cells (*r* = −0.703, *P* < 0.01), whereas it was positively correlated with neutrophils (*r* = 0.339, *P* < 0.05). Both in vivo and in vitro models confirmed the upregulated expression of *DUSP1* and the downregulated expression of *miR-429*.

**Conclusion:**

This study identified the ceRNA-based *AC079305/DUSP1* axis as a promising OS-related signature for IS. Immune infiltrating cells, especially mast cells, may exert a pivotal role in IS progression. Pharmacological agents targeting signatures, their receptors, or mast cells may shed a novel light on therapeutic targets for IS.

## 1. Introduction

Ischemic stroke (IS), accounting for over 80% of all clinical strokes globally [1], takes millions of lives every year and leaves survivors with permanent disabilities [2]. Those survivors are always characterized by symptoms such as hemiparesis, hemianaesthesia, aphasia, homonymous hemianopia, and dizziness, placing immense economic and psychological burdens on individuals and society [3]. At present, intravenous thrombolysis is regarded as a satisfactory treatment for IS; however, this therapy is limited by a narrow time window, specialized techniques, and high cost. Recently, RNA-sequencing (RNA-seq) has been reckoned as an indispensable technology to investigate gene intersection networks and identify candidate biomarkers for explorations of possible pathogenesis and novel therapeutic targets [4]. Accordingly, mining potential signature and therapeutic targets for IS through bioinformatics will compensate for our weaknesses in the early diagnosis, evaluation, and treatment of patients.

MicroRNAs (miRNAs), a class of small (20-23 nucleotides) single-stranded RNA without protein-coding ability, serve as important gene expression regulators and fine-tuners of a series of pathophysiological processes in multiple diseases including IS [5–7]. Long noncoding RNAs (lncRNAs) consist of long RNAs (>200 nucleotides) with no or limited protein-coding function. The aberrant expressions of lncRNAs can modulate pathological disorders of the central nervous system [8]. Mounting lines of studies have demonstrated that lncRNAs work as competing endogenous RNAs (ceRNAs) or natural miRNAs sponges to communicate with and coregulate each other, thereby repressing messenger RNA (mRNA) translation or promoting mRNA degradation [9]. Further exploration of this ceRNA crosstalk will benefit our understanding of the gene regulatory network in IS.

Oxidative stress (OS) serves as an essential initiator and propagator of neuroinflammation and neuron necrosis in IS, mainly caused by an imbalance between reactive oxygen species (ROS) production and consumption [10]. Overproduction of ROS (including glutathione, malondialdehyde, and peroxynitrite) communicates with innate immune receptors like NOD-like receptors and scavenger receptors, forming a stress microenvironment around fragile ischemic penumbra, which mediated inflammatory and immunocyte infiltration [11]. With such complex interactions, developing effective pharmacological agents based on a “one drug one target” scheme seems pretty difficult. Thus, exploring the unequivocal translational mechanism and specific signature of OS-related genes may provide novel clues for favorable treatment of IS.

In this study, GSE22255 and GSE110993 were used to identify differentially expressed genes (DEGs) between IS patients and controls. Gene Ontology (GO) and Kyoto Encyclopedia of Genes and Genomes (KEGG) enrichment analysis revealed their potential biological processes and pathways. Through four online database predictions, we successfully constructed an *AC079305*-mediated ceRNA network. The best differentially expressed oxidative stress-related gene (DEOSRG) was screened among DEGs, upregulated DEGs, OS-related genes, and the result of least absolute shrinkage and selection operator (LASSO) regression, whose ability to distinguish IS from controls was confirmed via the receiver operating characteristic (ROC) curve. Besides, CIBERSORT was adopted to investigate immune infiltration characteristics. In vivo rat and in vitro PC12 cells models were established to verify the best signature.

## 2. Materials and Methods

### 2.1. Data Sources

The RNA-seq profiles of 3 datasets (lncRNA cohort, GSE22255; mRNA cohort, GSE22255 and GSE140275; and miRNA cohort, GSE110993) were downloaded from the Gene Expression Omnibus (GEO) database (http://www.ncbi.nlms.nih.gov/geo). As a discovery dataset, GSE22255 enrolled 20 patients with IS and 20 sex- and age-matched controls with GPL570 (HG-U133_Plus_2) Affymetrix Human Genome U133 Plus 2.0 array platform [12]. GSE140275 was considered a validation dataset, consisting of 3 patients with IS and 3 healthy controls, whose platform was based on GPL16791 Illumina HiSeq 2500 [13]. GSE110993 was based on the GPL15456 Illumina HiScanSQ platform, including 20 IS patients and 20 matched controls, whose results were further validated (40 IS, 40 controls) and replicated (200 IS patients, 100 controls) [14]. See Table S1 in Supplementary Materials for more details about these 3 datasets. Since datasets were free from the publicly available databases, no ethics committee approval was required.

### 2.2. Data Preprocessing and Identification of DEmRNAs, DEmiRNAs, and DElncRNA

**Figure 1:**
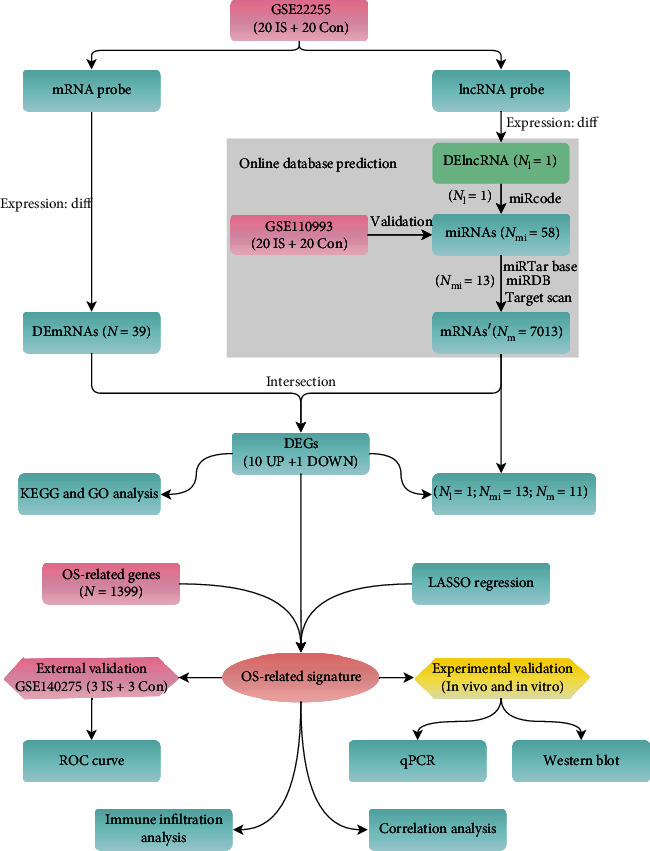
Entire working processes of the study. IS: ischemic stroke; Con: controls; DEGs: differently expressed genes.

After normalization and log2 conversion of raw data, we used the Linear Model for Microarray Data (LIMMA) method [15] in the R′ Bioconductor package for difference analysis. Genes with *P* < 0.05 and |log fold change (FC)| > 0.5 were marked as differentially expressed mRNAs, miRNAs, and lncRNAs (DEmRNAs, DEmiRNAs, and DElncRNAs), according to previously reported cut-off values by Tan et al. [16]. To enhance the veracity of differential analysis, we also conducted a predictive analysis through online databases. Briefly, DElncRNAs and DEmRNAs were firstly identified in GSE22255. Next, we put DElncRNAs into the miRcode database (v11, http://www.mircode.org/) to predict potential miRNAs matching with DElncRNAs. To obtain the downstream target mRNAs of the above miRNAs, three independent databases were used, including miRDB (v6.0, http://mirdb.org/), miRTarBase (v8.0, http://mirtarbase.mbc.nctu.edu.tw/php/index.php), and TargetScan (v7.2, http://www.targetscan.org/vert_72/). Herein, only when candidate target mRNAs' could interact with miRNAs from any two or three databases simultaneously could these mRNAs be grouped into mRNAs'. Finally, we sought the overlapped mRNAs between DEmRNAs and mRNAs' as the final DEmRNAs (corresponding to DEGs) for subsequent analyses. DEmiRNAs were identified in GSE110993. A heatmap plot and volcano plots were generated using R's pheatmap (v1.0.12) package. An upset Venn diagram was generated to reveal the online predictive process through the image GP website (http://www.ehbio.com/ImageGP/index.php/Home/Index/UpsetView.html). The entire working processes are shown in Figure 1.

### 2.3. Construction of a Triple ceRNA Network in IS

For the sake of elucidating the underlying transcriptional regulation mechanisms among genes, an lncRNA-miRNA-mRNA triple ceRNA network was constructed in IS, as in previously reported similar methods [17–19]. According to the four online databases (miRcode, miRDB, miRTarBase, and TargetScan), we integrated lncRNA-miRNA pairs and miRNA-mRNA pairs into the ceRNA network, which was visualized by Cytoscape (v3.8.0) [20]. The sequence of DElncRNA was fetched from the LNCipedia database (v5.2, https://lncipedia.org/), and the cellular localization of DElncRNA was extracted from the lncLocator database (v1.0, http://www.csbio.sjtu.edu.cn/bioinf/lncLocator/).

### 2.4. GO and KEGG Enrichment Analysis of DEGs

The “ggplot2 (v3.3.0),” “clusterProfiler (v3.14.3),” “org.Hs.eg.db (v3.10.0),” and “enrichplot (v1.6.1)” packages [21, 22] of R software were used to perform GO biological processes and KEGG pathway enrichment analysis of DEGs. Significantly enriched GO and KEGG terms were screened with thresholds of *P* < 0.05 and *Q* value < 0.05.

### 2.5. Selection of the Best DEOSRG

LASSO regression is a classical machine learning algorithm for dimension reduction with the use of a penalty parameter (*λ*). In this study, five-fold cross-validation (CV) was performed to select the optimal penalty parameter. We used the “glmnet (v4.1-2)” R package to remove genes that might overfit the model. Moreover, we sorted out 1399 OS-related genes from the GeneCards website (v5.7, https://www.genecards.org) using the key word “oxidative stress”, and the screening criteria was a relevance score ≥ 7 (see Table S2 in Supplementary Materials), according to previously reported methods [23]. A comprehensive strategy among the result of LASSO regression, OS-related genes, DEGs, and upregulated DEGs was adopted to select the best DEOSRG via the Venn tool (http://bioinformatics.psb.ugent.be/webtools/Venn/).

### 2.6. Evaluation and Validation of Discriminant Ability

To evaluate the discriminant ability of the best DEOSRG to distinguish IS from control groups, we utilized the “ROCR (v1.0-11)” R package to visualize the area under the ROC curve in the discovery dataset (GSE22255). Furthermore, we also validated this performance in an independent external dataset (GSE140275) in terms of the area under the ROC curve (AUCROC), 95% confidence interval (CI), specificity, and sensitivity.

### 2.7. Immune Infiltration Analysis

CIBERSORT, a versatile deconvolution algorithm, was performed to analyze the immune cell subset proportions using RNA expression profiles [24]. Herein, we obtained a matrix of 22 immune cell subsets in GSE22255 via “CIBERSORT (http://cibersort.stanford.edu, accessed on 03 February 2016)” and “parallel,” “e1071 (v1.7-8)”, and “preprocessCore (v1.48.0)” R packages. A bar plot displayed the percent of each immune cell. A heatmap depicting the abundance of 22 immune cells using “pheatmap (v1.0.12)” package was generated. The “vioplot (v0.3.7)” package was utilized to reveal the infiltrating levels between patients with IS and controls, while the “corrplot (v0.90)” package was carried out to reflect the relationship of immune cell subsets via a correlation heatmap. *P* < 0.05 was determined to be statistically significant.

### 2.8. Correlation Analysis

To investigate the relationship between the best DEOSRG and immune cell subsets, we applied Spearman correlation analysis using R's “tidyverse (v1.3.1),” “ggsci (v2.9),” and “ggplot2 (v3.3.0)” packages. What is more, we estimated the association of the best DEOSRG with the matching lncRNA. *P* < 0.05 was considered statistically significant.

### 2.9. Animals and Cerebral I/R In Vivo Model

Male SD rats (240-270 g) were purchased from the Laboratory Animal Centre of Xi'an Jiaotong University and were housed in a standard animal facility with 12 h light/dark cycle (25°C room temperature). Their water and food were available ad libitum. The protocol was approved by the Laboratory Animal Ethics Committee of Xi'an Jiaotong University (Xi'an, Shaanxi, China). Before developing an occlusion of middle cerebral artery (MCAO) in vivo model according to the previously reported method [25], rats were deprived of food and water for 8 h. Briefly, the right carotid sheath was exposed, the common carotid artery (CCA) was separated, and the proximal end of CCA was ligated under the anesthesia condition. After separating the internal carotid artery (ICA) and external carotid artery (ECA), the ECA was ligated. The distal end of CCA was clipped with a nontraumatic artery clamp, and a small incision was made with ophthalmic scissors. Then, the middle cerebral artery (MCA) was effectively blocked with a nylon suture coated with poly-L-lysine (Beijing Cinontech, China) and fixed with a preplaced 5.0 silk suture. 90 minutes after MCAO, the nylon suture was removed to achieve reperfusion and the wound was closed, which was also named cerebral ischemia/reperfusion (I/R) model. After reperfusion for 24 h, the ischemic penumbra was taken according to the previously described method [26]. Rats were allocated into the following groups: Sham group and I/R group. Rats in the Sham group suffered from the same invasion without true blood vessel occlusion.

### 2.10. Cell Culture and OGD/R In Vitro Model

PC12 cells, a rat pheochromocytoma cell line, were obtained from the Cell Resource Center of Shanghai Institute of Biological Science, Chinese Academy of Sciences. PC12 cells were cultured in DMEM medium supplemented with 10% fetal bovine serum (FBS), 100 U/ml penicillin, and 100 *μ*g/ml streptomycin (no. C100C5, NCM Biotech Co. Ltd., China). Cultures were maintained at 37°C with 5% CO_2_ in a humidified cell incubator. The subsequent analyses were conducted when the density of the cell cultures reached 60%–80%. An oxygen-glucose deprivation/reperfusion (OGD/R) in vitro model was established to mimic cerebral I/R. Briefly, PC12 cells were washed 2-3 times with PBS. Then, they were cultivated with glucose-free and serum-free DMEM medium (no. 11966025, Gibco, USA) combined with three gas (94% N_2_, 5% CO_2_, and 1% O_2_) in culture environment at 37°C for 4 h. After that, the cells were restored to normal condition for 24 h as reperfusion. Cells were allocated into the following groups: Control group and O/R group.

### 2.11. RNA Extraction and Quantitative PCR

Total RNA samples were isolated from cells or frozen brain tissue with RNAiso Plus (no. SD1412, Takara, Japan), and 1 *μ*g of total RNA was used for cDNA synthesis by using the reverse transcription kit (no. G490, abm, Canada). For synthetic cDNA of *miR-429*, the miRNA First-Strand cDNA Synthesis Kit (no. B532453, Sangon Biotech, China) was used. qPCR was conducted to amplify the synthetic cDNA templates using BlasTaq™ 2X qPCR Master Mix (no. G891, abm, Canada) according to the manufacturer's instructions. The gene expression levels were normalized to GAPDH or U6 via the 2^−ΔΔC^ method. The primers used are shown in Table S3.

### 2.12. Western Blot

Frozen brain tissue or collected cells were lysed by RIPA lysis containing a protease inhibitor and PMSF (no. PL001, ZHHC, China). The lysate mixtures were centrifuged at 12000 rpm (4°C) for 10 min. The supernatants from the centrifugal tube were protein samples. Collected proteins and took a small portion of them to quantify concentrations by using a BCA kit (no. P0010, Beyotime, China). The remaining proteins were mixed with 4X loading buffer, boiled, and denatured for 10 min. The equal proteins were transferred to 0.45 *μ*m PVDF membranes after complete separation with 10% SDS-PAGE electrophoresis. The membranes were blocked with protein free rapid blocking buffer (no. PS108P, Epizyme, China) and incubated overnight (4°C) with the primary antibodies against DUSP1 (no. 381573, 1 : 1000, rabbit polyclonal, ZEN-BIOSCIENCE, China), while an anti-*β*-actin antibody was used as the internal loading control. The next day, the membranes were incubated with the secondary antibody (room temperature) for 1 h and then washed with TBST for 10 min 3 times on a shaking incubator. Protein bands were visualized using an imaging system (Bio-Rad, Francisco, USA) after incubation with ECL chemiluminescence (no. MI00607A, Mishushengwu, China). Using ImageJ software (v1.46r, National Institutes of Health, USA), analyzed the relative protein expression levels.

### 2.13. Statistical Analyses

All statistical analyses were conducted by using R software (v4.1.0, https://www.r-project.org/) and GraphPad Prism software for Windows (v8.0, San Diego, California, USA). Differential expression analysis was performed with the cut-off thresholds of *P* < 0.05 and |*logFC*| > 0.5. The upregulated expression was defined as logFC > 0, while the downregulated expression was defined as logFC < 0. The experimental data were presented as mean ± SEM. For normally distributed variables, Student's *t*-test was used to compare the differences between two groups, while the Mann-Whitney *U* test was used for abnormally distributed variables. *P* < 0.05 was considered a significant difference.

## 3. Results

### 3.1. Identification of DEmRNAs, DEmiRNAs, and DElncRNA in IS

**Figure 2:**
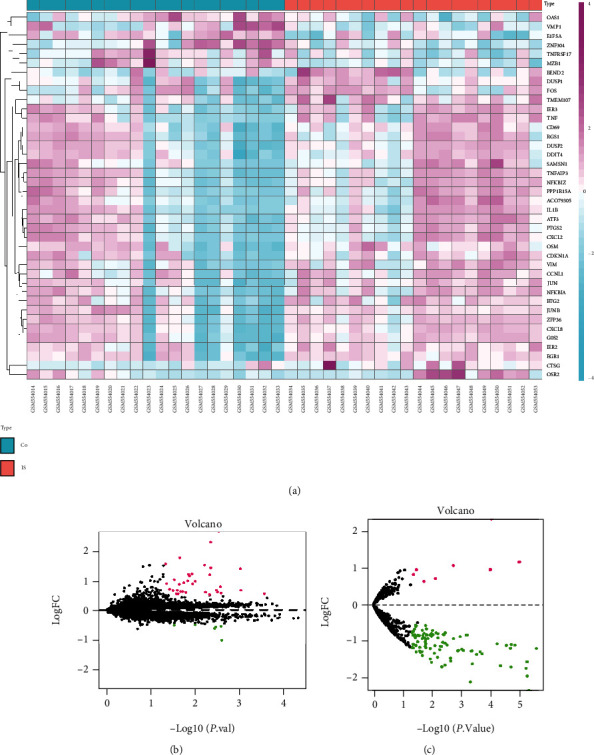
Difference analysis for mRNAs, lncRNAs, and miRNAs. The identification criteria: *P* < 0.05 and |*logFC*| > 0.5. (a) Heatmap plot showing 1 DElncRNA and 39 DEmRNAs in GSE22255. Pink represents upregulated genes, and blue indicates downregulated genes. Co: controls; IS: ischemic stroke. (b, c) Volcano plots for DEmRNAs and DEmiRNAs. (b) 39 DEmRNAs (33 upregulated and 6 downregulated) in GSE22255. (c) 119 DEmiRNAs (8 upregulated and 111 downregulated) in GSE110993. Black dots represent genes equally represented between IS patients and controls. Pink and green dots represent upregulated genes and downregulated genes, respectively.

In GSE22255, a total of 39 DEmRNAs were directly identified, while *AC079305* was the only DElncRNA in patients with IS compared with controls (Figure 2(a)). Of the 39 DEmRNAs, 33 were upregulated and 6 were downregulated (Figure 2(b)). In GSE110993, 8 upregulated (e.g., *miR-367* and *miR-17*) and 111 downregulated DEmiRNAs (e.g., *miR-429* and *miR-206*) were identified (Figure 2(c)).

**Figure 3:**
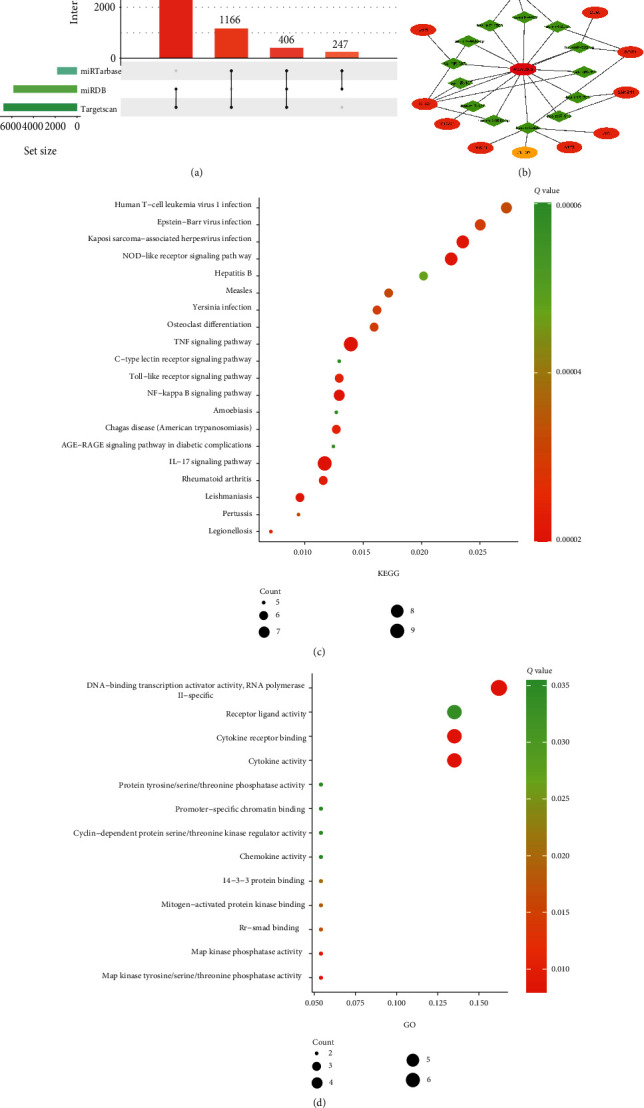
Construction of a triple ceRNA network and functional enrichment analysis. (a) Upset Venn diagram of online prediction analysis. Three online databases—miRTarBase, miRDB, and TargetScan—are used to predict target mRNAs of miRNAs, and the filtrated condition of target mRNAs is that they must be interacted by any two or three databases. (b) The triple ceRNA network in IS. The octagon denotes lncRNA, diamond denotes miRNAs, and ellipse denotes mRNAs (orange represents upregulated, and yellow represents downregulated). (c) Top 20 KEGG enrichment pathways for 11 DEGs. (d) GO biological processes for 11 DEGs.

As shown in Figures 1, 58 potential miRNAs could combine with *AC079305*. According to miRDB, miRTarBase, and TargetScan databases, merely 13 miRNAs of the predicted 58 miRNAs could match with 7013 downstream mRNAs' (Figure 3(a)). After taking an intersection, we focused on 11 remaining DEGs (including *JUN*, *DUSP1*, *ZNF304*, *BTG2*, *CD69*, *CDKN1A*, *ATF3*, *PTGS2*, *SAMSN1*, *DDIT4*, and *OSM*), which shared genes between 7013 target mRNAs' and 39 DEmRNAs.

### 3.2. Construction of the ceRNA Network

To illuminate the underlying transcriptional regulatory signatures of IS, a triple ceRNA network was constructed. As presented in Figure 3(b), *AC079305* was the only identified DElncRNA, which might play a crucial role in the network. There were 13 miRNA nodes and 11 mRNA nodes in the *AC079305*-mediated ceRNA network. The 33 edges represented 13 lncRNA-miRNA and 20 miRNA-mRNA interactions.

### 3.3. GO and KEGG Enrichment Analysis of DEGs

KEGG enrichment results showed that the 11 DEGs involved in the ceRNA network were predominantly enriched in the following pathways: tumor necrosis factor (TNF) signaling pathway, interleukin-17 (IL-17) signaling pathway, NOD-like receptor signaling pathway, and Toll-like receptor signaling pathway (Figure 3(c)). The top 20 KEGG enrichment analyses are displayed in Table S4 in Supplementary Materials. GO enrichment revealed that 11 DEGs were significantly enriched in biological processes, such as receptor ligand activity, cytokine receptor binding, cytokine activity, MAP kinase phosphatase activity, and MAP kinase tyrosine/serine/threonine phosphatase activity (Figure 3(d)). More details of per GO term are shown in Table S5 in Supplementary Materials.

### 3.4. Selection of the Best DEOSRG in IS

**Figure 4:**
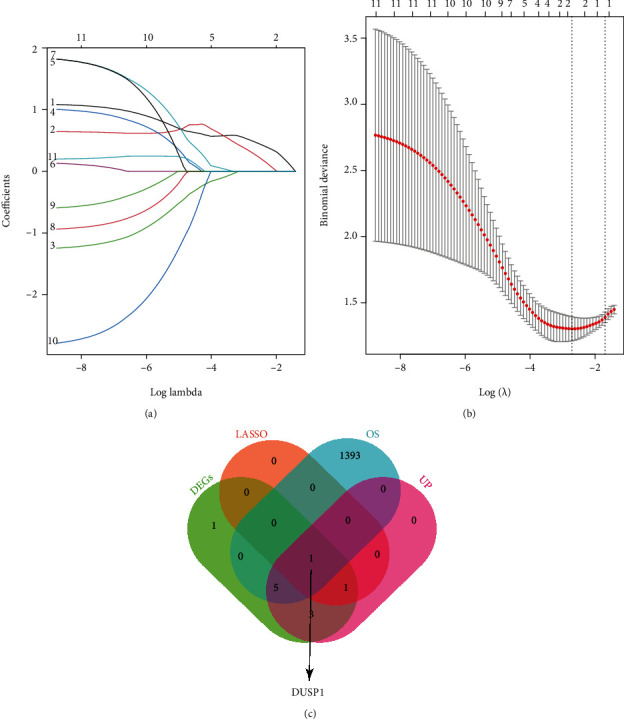
Selection of the best DEOSRG in IS via the comprehensive strategy. (a, b) LASSO regression screening for candidate genes. (a) LASSO coefficient profiles for 11 differentially expressed genes. (b) Binomial deviance profiles for 11 differentially expressed genes. (c) A 4-set Venn diagram showing the comprehensive strategy among DEGs (green circle), LASSO regression (orange circle), OS-related genes (blue circle), and upregulated DEGs (pink circle). As shown, *DUSP1* is the best DEOSRG. DEOSRG: differentially expressed oxidative stress-related gene.

In dataset GSE22255, totally 11 DEGs were defined. Of those DEGs, 10 were sorted out to be upregulated genes. To select the best DEOSRG in IS, we adopted a comprehensive strategy among the result of LASSO regression, DEGs, upregulated DEGs, and OS-related genes. The LASSO regression result selected two possible signatures (seed = 100, CV = 5): *DUSP1* and *JUN* (Figures 4(a) and 4(b)). Then, the Venn diagram showed that *DUSP1* was the best DEOSRG (Figure 4(c)).

### 3.5. Evaluation and Validation of the Discriminant Ability of *DUSP1*

**Figure 5:**
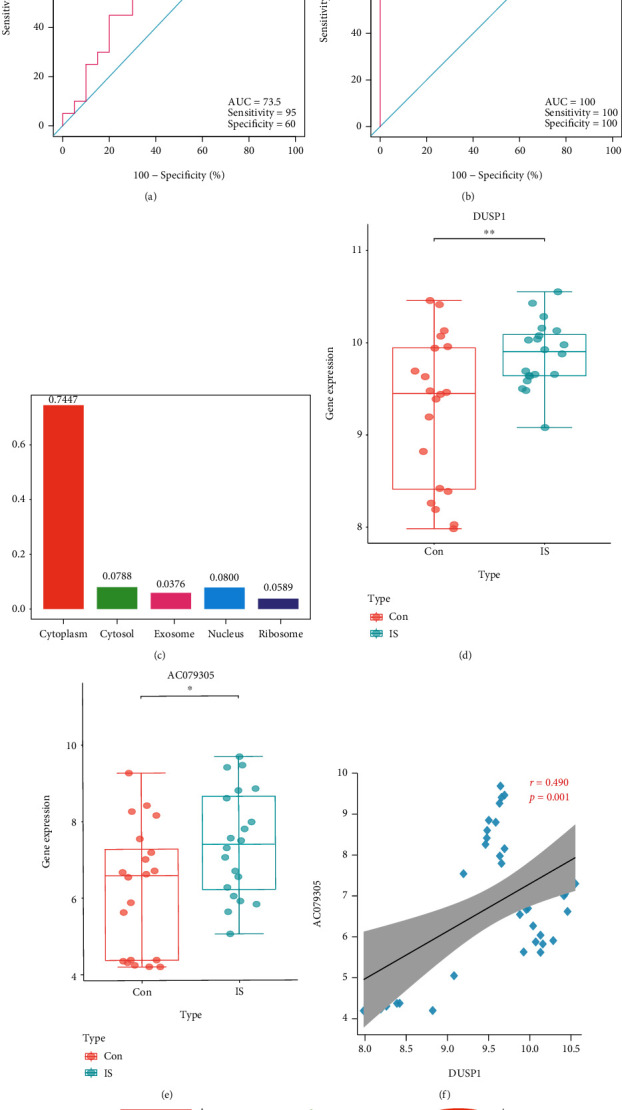
ROC analysis and OS-related *AC079305/DUSP1* axis analysis. (a) ROC analysis for *DUSP1* in the discovery dataset. (a) ROC analysis for *DUSP1* in the validation dataset. (c) The cellular localization of *AC079305* is based on lncLocator. (d) The expression patterns of *DUSP1* in IS patients and controls. (e) The expression patterns of *AC079305* in IS patients and controls. (f) The Spearman correlation analysis between *DUSP1* and *AC079305* in IS. (g) Diagram of the *AC079305/miR-429/DUSP1* axis. ROC: receiver operating characteristic; OS: oxidative stress.

After that, we used the ROC curve to evaluate the discriminant ability of *DUSP1* in the GSE22255 dataset. As shown in Figure 5(a), the AUCROC was 73.5% (95%CI = 57.02-89.98, sensitivity = 95%, and specificity = 60%). To further verify the diagnostic performance of *DUSP1* for distinguishing patients with IS from controls, we validated it in an external GSE140275 dataset, which reached a higher AUCROC of 100.0% (*95*%CI=100.00-100.00, sensitivity = 100%, and specificity = 100%) (Figure 5(b)). Collectively, the above results suggested that *DUSP1* was a reliable and promising signature for predicting patients with IS.

### 3.6. Identification of the OS-Related *AC079305/DUSP1* Axis in IS

According to the ceRNA network, *miR-429* was the hub gene-linked *AC079305*/*DUSP1* axis with downregulated expression level (logFC = −0.644, *P* < 0.05). On the contrary, *AC079305* and *DUSP1* were both upregulated (*AC079305*: logFC = 1.202, *P* < 0.05; *DUSP1*: logFC = 0.603, *P* < 0.05). Unfortunately, it is difficult to obtain *miR-429* expression levels of each sample in GSE110993. Therefore, the role of the *AC079305*/*DUSP1* axis was concentrated. We used a box diagram to visualize their expression levels between patients with IS and controls (Figures 5(d) and 5(e)). What is more, Figure 5(c) showed that *AC0790305* was particularly distributed in the cytoplasm. Additionally, expression correlation analysis confirmed that *AC079305* was positively related to *DUSP1* (*r* = 0.490, *P* < 0.01) (Figure 5(f)). Taken together, these data indicated that *AC079305* might act as a ceRNA to prompt the expression level of *DUSP1* via sponging *miR-429* in the cytoplasm, which might be involved in oxidative stress during the occurrence and development of IS.  Figure 5(g) displayed the interaction of the *AC079305/miR-429/DUSP1* axis. Hence, the *AC079305*/*DUSP1* axis in the ceRNA network was selected as a robust OS-related signature to distinguish IS patients from controls.

### 3.7. Immune Infiltration Analysis

**Figure 6:**
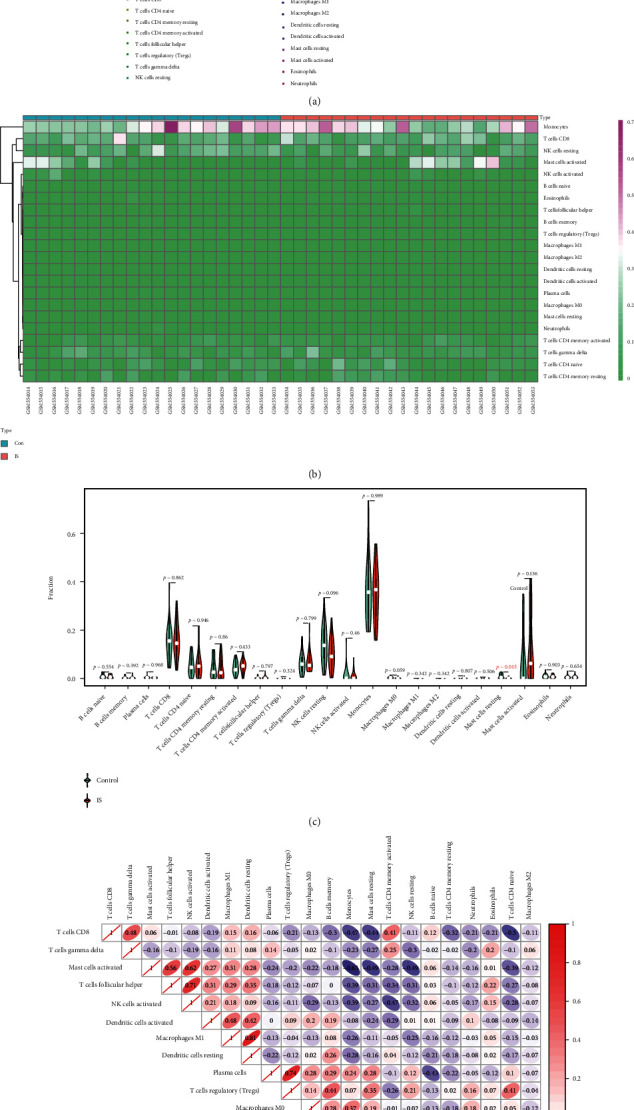
Immune infiltration characteristics between IS patients and controls. (a) The relative percent of 22 immune cell subsets in a bar plot. (b) Heatmap of 22 immune cell subsets. (c) Infiltrating difference of immune cells between IS patients and controls in the violin plot. The red mark represents a significant infiltrating difference. (d) Correlation heatmap for 22 immune cell subsets. Red is marked as a positive correlation, and purple is marked as a negative correlation. And the darker the color, the stronger the correlation index.

A series of immunological episodes from peripheral immune cell invasion and microglial activation to the secretion of proinflammatory factors have been reported to be triggered after IS [27]. Hence, we analyzed the immune infiltration characteristics between patients with IS and controls using the CIBERSORT method. The bar plot and heatmap depicted the relative percent of diverse immune cell subsets in each sample (Figures 6(a) and 6(b)). Compared with controls, resting mast cells presented a lower infiltrating level in patients with IS, as shown in the violin plot (Figure 6(c)). A correlation heatmap of 22 immune cell subsets displayed that activated NK cells were positively related not only to activated mast cells (*r* = 0.62) but also to follicular helper T cells (*r* = 0.71), M1 macrophages were positively related to resting dendritic cells (*r* = 0.81), and plasma cells were positively related to regulatory T cells (*r* = 0.74). Resting mast cells were positively related to naive CD4 T cells (*r* = 0.47), monocytes (*r* = 0.50), and memory B cells (*r* = 0.35), whereas they were negatively related to CD8 T cells (*r* = −0.44) and activated mast cells (*r* = −0.49). And monocytes were negatively correlated with activated mast cells (*r* = −0.63) (Figure 6(d)).

### 3.8. Correlation between Immune Infiltration and Expression of *DUSP1* in IS

**Figure 7:**
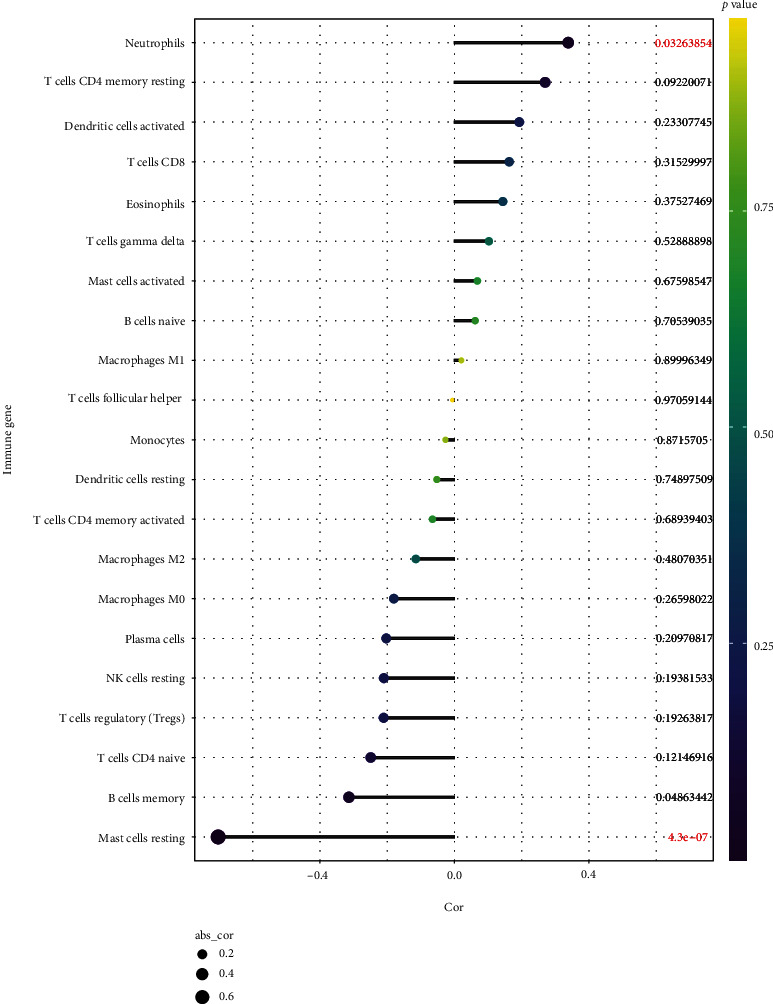
Spearman correlation between immune cell subsets and *DUSP1*. The color of dots denotes the *P* value. The size of dots denotes the strength of correlation.

To further elucidate the correlation between *DUSP1* and diverse immune cell subsets, Spearman correlation analysis was conducted further. The results suggested that *DUSP1* was positively correlated with neutrophils (*r* = 0.339, *P* < 0.05), while it was negatively correlated with resting mast cells (*r* = −0.703, *P* < 0.01) (Figure 7). See Table S6 in Supplementary Materials for more details. To conclude, *DUSP1* was closely associated with resting mast cells, indicating that resting mast cells might play an essential part during oxidative stress in IS progression.

### 3.9. *miR-429* and *DUSP1* Expressions in In Vitro and In Vivo Models

**Figure 8:**
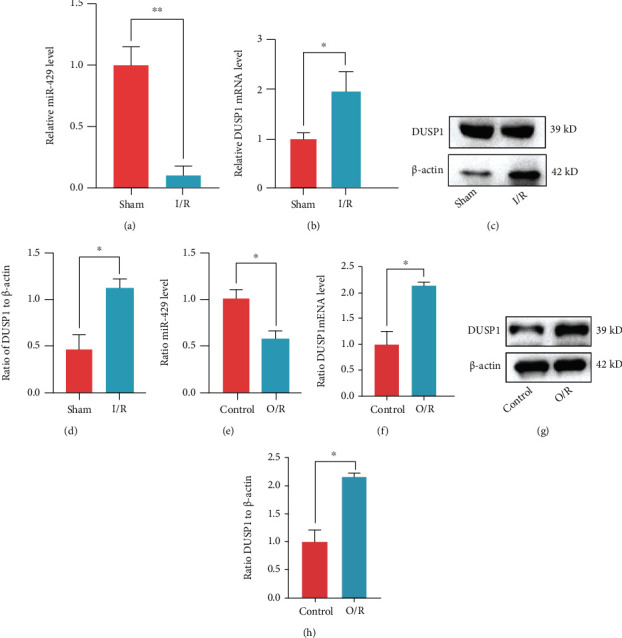
*miR-429* and *DUSP1* expression in vitro and in vivo models. (a) The mRNA level of *miR-429* in rat brain tissues. (b) The mRNA level of *DUSP1* in rat brain tissues. (c, d) The protein level of DUSP1 in rat brain tissues. (e) The mRNA level of *miR-429* in PC12 cells. (f) The mRNA level of *DUSP1* in PC12 cells. (g, h) The protein levels of DUSP1 in PC12 cells. ^∗^*P* < 0.05, ^∗∗^*P* < 0.01, compared to the sham/control group.

The in vivo model showed that compared with the Sham group, the expression of *miR-429* in the brain tissue from rats in the I/R group was significantly decreased (Figure 8(a)). However, *DUSP1* expression in both groups yielded an opposite trend to *miR-429*, at both mRNA (Figure 8(b)) and protein levels (Figures 8(c) and 8(d)). Simultaneously, the in vitro model showed that the *miR-429* expression in the PC12 O/R group was also notably lower than that in the Control group (Figure 8(e)). In contrast, the expression of *DUSP1* in the O/R group was obviously increased compared with that in the Control group, at both mRNA (Figure 8(f)) and protein levels (Figures 8(g) and 8(h)). These results suggested that the *miR-429* might inhibit the expression of *DUSP1*.

## 4. Discussion

As a disease increases the risk and incidence of irreversible disability and death, IS still preserves a tremendous challenge for the worldwide healthy system. Although various emerging molecules and diagnostic techniques have been developed, they have not sufficiently improved the early diagnosis, treatment, and prognosis of patients with IS. Increasing evidence has confirmed that oxidative stress participated in the fundamental pathologic progression of IS. However, very few studies have focused on OS-related signature as a ceRNA mechanism and immune infiltration as a characteristic of IS progression, which might be conducive to early diagnosis and precise therapy of patients with this disease. To this end, we totally identified 11 DEGs for further exploration. An *AC079305*-mediated triple ceRNA network was attempted to construct. Interestingly, we selected the best OS-related gene—*DUSP1*—based on the comprehensive strategy with the excellent discriminant ability for IS. The CIBERSORT algorithm showed immune infiltration characteristics in IS. As far as we know, the current study firstly explored the involvement of *DUSP1* in oxidative stress, its ceRNA regulatory mechanism, and immune infiltration correlation during IS development.

The Venn intersection among the result of LASSO regression, DEGs, uprelated DEGs, and OS-related genes constructed the best feature signature—*DUSP1*. Dual-specificity phosphatase 1 (*DUSP1*), as one of the dual-specificity phosphatase family members, encodes MKP1 protein. The most classical effect of MKP1 is involved in regulating cellular biological processes, such as metabolism, death, differentiation, and proliferation by dephosphorylating mitogen-activated protein kinase (MAPK) including JNK specifically in the cytoplasm or nucleus [28]. However, the focus of this study was to explore the role of *DUSP1* in oxidative stress during the progression of IS. Several lines of evidence indicated the involvement of *DUSP1* in oxidative stress processes at nonstroke fields. For instance, Liu et al. [29] clarified that p53 was a transcriptional regulator of *DUSP1* in signaling the cellular response to oxidative stress. Similarly, Wang et al. [30] reported that E2F-1 could bind to a variant of its consensus sequence in the promoter of the *DUSP1*, and oxidative stress increased their binding. Oxidative stress modified E2F-1 by acetylation and promoted E2F-1 binding to a palindrome motif for transcriptional upregulation of *DUSP1*. Kim et al. [31] demonstrated MKP-1, encoded by *DUSP1*, contributing to oxidative stress-induced apoptosis via inactivation of ERK1/2 in SH-SY5Y cells. In stroke-related field, evidences on *DUSP1* were mostly limited to gene expression for IS diagnosis, which rarely involved a transcriptional regulatory mechanism and immune infiltration. Adamski et al. [32] reported that seven transcripts, including *DUSP1* from while blood samples, presented a optimal accuracy for ischemic stroke classification. Consistent with our findings, a higher expression of *DUSP1* was noted in patients with IS than in controls. Zhang et al. [3] also concluded that *DUSP1* upregulation participated in the pathogenesis of IS by bioinformatics analysis, but when they validated it with blood samples from five patients with IS and five controls by qRT-PCR, the opposite results were observed. They attributed this inconsistency to a small sample size. Our in vitro and in vivo experiments showed the overexpression of *DUSP1* in IS, which kept in pace with bioinformatics results. An in vitro experiment demonstrated that overexpressed MKP1 exerted its neuroprotective effect through inhibiting neuronal death and the JNK signaling pathway [33]. Wang et al. [34] confirmed that MKP1 served to limit the inflammatory responses through inactivating p38 and JNK. Additionally, *DUSP1* was also been reported to play promoter and suppressor roles in atherosclerosis [35, 36]. Compared with the above studies, the advantages of our study lie in not only verifying *DUSP1* as a robust signature for identifying IS but also exploring the ceRNA regulatory mechanism and immune infiltration microenvironment of IS. We supposed that *DUSP1* might participate in the oxidative stress process during IS through dephosphorylation of MAPK-related pathways. Combining our findings with previous studies, the function and role of *DUSP1* in IS should be the center of subsequent investigations.

The *miR-429*, as the upstream target of *DUSP1*, belongs to the miR-200 family. Several previous studies have noted that *miR-429* regulates proliferation, apoptosis, and invasion in cancers [37, 38]. Consistent with our results, the *miR-200* family was reported to be upregulated after cerebral ischemic preconditioning, playing a neuroprotective role by suppressing prolyl hydroxylase 2 expression [39]. As for *AC079305*, few studies have investigated its role in ischemic stroke. PubMed Gene defined *AC079305* as a complete sequence of Homo sapiens BAC clone RP11-483K11. Given that the crosstalk of ceRNA only exists in the cytoplasm, we also performed subcellular localization analysis of *AC079305*, which confirmed that it exerted regulatory functions predominantly in the cytoplasm. Moreover, we found relatively significant correlation between *AC079305* and *DUSP1*. In this study, bioinformatics results showed that *AC079305*/*DUSP1* was upregulated; opposite to them, *miR-429* was downregulated. The in vivo and in vitro results confirmed the upregulated expression of *DUSP1* and downregulated expression of *miR-429*. Since the sequence information of *AC079305* derived from rat was not available at present in the NCBI gene sources, we could not verify its expression in animal or cell experiments. Based on the ceRNA mechanism, we speculated that *AC079305* improves the expression level of *DUSP1* via sponging *miR-429* to aggravate oxidative stress during IS progression. More randomized controlled clinical trials with large samples are needed to validate this key OS-related axis in IS.

Accumulative evidence supported that IS led to the inflammatory response in the entire body, not just in the peri-infarct region [40]. On the one hand, ROS and immune dysfunction generated in ischemic brain parenchyma may spill into the circulating blood activating systematic immune-inflammatory response [41]. On the other hand, increasing oxidative mediators break the blood brain barrier inducing the infiltration of blood-deprived immune cells (including T cells, B cells, macrophages, and dendritic cells), further exacerbating neurotoxicity and neuroinflammation [42]. Meng et al. [43] found that double-negative T cells were gradually increased in a time-dependent manner based on human study or MCAO mice. However, our findings observed that significantly fewer resting mast cells were found in the peripheral blood of patients with IS compared with controls, whereas no significant difference was found in T cells. This discrepancy may be explained by the fact that a relatively small sample size was applied in our analysis. Mast cells consist in different brain regions in mammals, such as the hippocampus, meninges, cortex, and thalamus. In the central nervous system (CNS), mast cells not only participate in the immune response as the first immune sentinel cells but also communicate with neurons, microglia, and glia. An increasing body of evidence supports a contribution of mast cells in the pathogenesis of stroke. After an interruption of cerebral blood flow, mast cells are able to sense alerting signals from the ischemic brain and become activated [44]. Through amplifying neuroinflammation and releasing gelatinases, activated mast cells aggravate vasogenic edema and hemorrhagic transformation, thereby contributing to poor prognosis for IS patients [44–46]. Mattila et al. [47] clarified that the degree of brain edema was related to gelatinase activity in the ischemic tissue, and activated mast cells exactly secreted gelatinase-positive granules. Some other scholars supported that parenchymal mast cells might not play a pivotal role in ischemic stroke. For instance, Arsene et al. [48] found mast cells absent in the penumbra surrounding the necrotic brain tissues of deceased patients with ischemic stroke based on a human-derived immunohistochemical study. In a MCAO mouse model, Arac et al. [49] provided evidence that meningeal mast cells worsened infiltration of granulocytes and macrophages, brain swelling, and infarct size. Although the function of mast cells had been extensively studied in CNS, few studies reported the relationship between *DUSP1* and mast cells in ischemic brain tissue. The spearman correlation result showed that *DUSP1* was negatively correlated with resting mast cells, with the correlation coefficient (*r*) of -0.704. In 2001, Kassel et al. [50] firstly reported the function of *DUSP1* in mast cells in allergy. Specifically, glucocorticoid-induced increase in the *DUSP1* expression resulted in the dephosphorylation of MAPK, thereby playing an anti-inflammatory effect in mast cells. Dellinger et al. [51] reported similar molecular mechanism of *DUSP1* in lung mast cells with the induction of myo-inositol modified 70-carbon fullerenes (an inhaled corticosteroid analogue). Although the above evidences supported our result, how this relationship works in ischemic brain tissue remains to be elucidated. As oxidative stress and immune inflammation are involved in IS, it is plausible to introduce antioxidant or anti-inflammatory agents into the management of IS patients, but studies attempted to find novel and effective drugs which are absent in IS.

In our GO results, a set of MAPK-associated biological processes were found to be enriched, such as MAP kinase phosphatase activity and MAP kinase tyrosine/serine/threonine phosphatase activity. MAPKs, inactivated by the DUSP family, have broad substrate specificity targeting p38 MAPK, stress-activated protein kinase/JNK, and so on, indicating that MAPKs regulate diverse cellular responses. This once again confirmed that DEGs including *DUSP1* were closely related to MAPK. Under the stimulation with oxidative stress, heat shock, or other factors, several MAKPs are easily induced affording immediate-early proteins [52]. Jeanneteau et al. [53] reported that MKP1 mediated axonal remodeling by neurotrophin brain-derived neurotrophic factor in a spatiotemporal way. KEGG enrichment results revealed the DEGs primarily involved in the IL-17 signaling pathway, NOD-like receptor signaling pathway, and Toll-like receptor signaling pathway. After ischemic stroke, IL-17 was activated in the damaged brain areas and broke the blood brain barrier via producing massive reactive oxygen species, thereby contributing to neurodegeneration [54]. NOD-like and Toll-like receptors were reported to stimulate inflammatory responses in IS; some pharmacological agents targeting these receptors have been investigated specifically [55, 56]; however, few of these agents translate into clinical practices despite some inspiring discoveries.

The present study also has several limitations. First, the specific binding affinity among genes should be experimentally and clinically investigated to substantiate claims. Second, the CIBERSORT algorithm has an inherent limitation to overrate or underrate cell subsets despite a relatively lower evaluation bias compared to other algorithms. Moreover, further functional validation of the *AC079305*/*DUSP1* axis in more randomized controlled clinical trials with large samples is warranted.

## 5. Conclusions

In summary, this study established an overexpressed *AC079305*/*DUSP1* axis as robust and promising oxidative stress-related signatures in IS. Immune cells, especially mast cells, are involved in the pathogenesis of IS; pharmacological agents targeting those molecules, their receptors, or cerebral mast cells may serve as a potential neuroprotective strategy for patients with IS.

## Data Availability

The datasets generated and/or analyzed during the current study are available in the GEO database (https://www.ncbi.nlm.nih.gov/) (GEO profiles: GSE22255, GSE110993, and GSE140275) and GeneCards website (https://www.genecards.org). And they are also available from the corresponding author, upon reasonable request.
