# Supplementary material for: Investigating the AC079305/DUSP1 Axis as Oxidative Stress-Related Signatures and Immune Infiltration Characteristics in Ischemic Stroke
Source: Oxid Med Cell Longev. 2022 Jun 14;2022:8432352. doi: 10.1155/2022/8432352 (PMC9213160; doi:10.1155/2022/8432352)
Supplement: Supplementary Materials — The supplementary material for this article can be found in the Supplementary Materials file. [file 8432352.f1.docx]

**Supplementary Materials**

**Table S1 Detailed information of the studied RNA-Seq profiles.**

| **Dataset** | **Platform** | **Control** | **IS** | **Contact** | **Country** | **Last update date** | **Samples** | **Application** |
| --- | --- | --- | --- | --- | --- | --- | --- | --- |
| GSE22255 | GPL570 | 20 | 20 | Sofia A Oliveira ^[1]^ | Portugal | Mar/25/2019 | Peripheral blood mononuclear cells | Identification for DEmRNAs and DElncRNAs |
| GSE110993 | GPL15456 | 20 | 20 | Bernd Henrik Northoff ^[2]^ | Germany | Mar/16/2020 | Peripheral blood | Identification for DEmiRNAs |
| GSE140275 | GPL16791 | 3 | 3 | Shenghua Li ^[3]^ | China | Jun/29/2020 | Circulating blood | Validation for DEmRNAs |

Abbreviation: IS, ischemic stroke.

References:

[1] Krug T, Gabriel JP, Taipa R, et al. TTC7B emerges as a novel risk factor for ischemic stroke through the convergence of several genome-wide approaches. J Cereb Blood Flow Metab 2012 Jun;32(6):1061-72.

[2] Tiedt S, Prestel M, Malik R, et al. RNA-Seq Identifies Circulating miR-125a-5p, miR-125b-5p, and miR-143-3p as Potential Biomarkers for Acute Ischemic Stroke. Circ Res 2017 Sep 29;121(8):970-980.

[3] Li S, Chen L, Xu C, et al. Expression profile and bioinformatics analysis of circular RNAs in acute ischemic stroke in a South Chinese Han population. Sci Rep 2020 Jun 23;10(1):10138.

**Table S2 Summary of oxidative stress-related genes extracted from the GeneCards with a relevance score ≥ 7 (n=1399).**

| **Gene Symbol** | **Relevance score** | **Gene Symbol** | **Relevance score** | **Gene Symbol** | **Relevance score** | **Gene Symbol** | **Relevance score** | **Gene Symbol** | **Relevance score** |
| --- | --- | --- | --- | --- | --- | --- | --- | --- | --- |
| KIT | 7 | DECR1 | 8.25 | GFM2 | 9.92 | HTR2C | 12.35 | APOA1 | 16.69 |
| NDUFA13 | 7.01 | NR1H2 | 8.26 | SETX | 9.92 | ENO1 | 12.36 | CYGB | 16.76 |
| MAPK13 | 7.01 | HBEGF | 8.27 | ADAMTS13 | 9.93 | LAMP2 | 12.37 | CCND1 | 16.8 |
| TNFSF4 | 7.02 | AKAP9 | 8.28 | GHRL | 9.94 | ITGB1 | 12.37 | PDHA1 | 16.81 |
| LEPQTL1 | 7.02 | MIR34C | 8.3 | KCNMA1 | 9.94 | SERPINA1 | 12.37 | ASS1 | 16.88 |
| MIR185 | 7.03 | MSR1 | 8.3 | MIR125A | 9.95 | S100B | 12.37 | LRRK2 | 16.9 |
| ALS3 | 7.04 | RPA1 | 8.31 | H2AX | 9.96 | LOC110973015 | 12.37 | HTR2A | 16.92 |
| ALS7 | 7.04 | RCAN1 | 8.32 | FOXP3 | 9.96 | REST | 12.4 | DRD2 | 16.94 |
| OXTR | 7.05 | FTH1 | 8.32 | KRT8 | 9.97 | PIK3R1 | 12.4 | SLC6A3 | 16.96 |
| DHFR | 7.05 | CYP11A1 | 8.32 | PLA2G2A | 9.98 | CDC42 | 12.4 | MAPKAPK2 | 17.07 |
| ACACA | 7.05 | CHGA | 8.33 | BRF2 | 9.98 | CXCL12 | 12.4 | ARG1 | 17.08 |
| GPX8 | 7.05 | PLCB1 | 8.33 | MIR200B | 9.98 | DAO | 12.41 | GGT1 | 17.1 |
| BGLAP | 7.06 | SENP3 | 8.33 | FKRP | 9.98 | BAK1 | 12.41 | SLC25A4 | 17.11 |
| TAZ | 7.07 | RRM2B | 8.33 | ADPRS | 9.99 | HCRT | 12.41 | NPY | 17.12 |
| MAPK12 | 7.08 | PDK1 | 8.34 | H4-16 | 9.99 | ADORA2A | 12.43 | GBA | 17.2 |
| YBX1 | 7.08 | SNCB | 8.34 | ALAD | 9.99 | MTHFR | 12.46 | BMP6 | 17.24 |
| SFXN4 | 7.09 | MIR107 | 8.34 | MIR181A1 | 10 | CSF3 | 12.47 | HSPD1 | 17.28 |
| SLC4A1 | 7.09 | MIR181C | 8.36 | MT-ND4 | 10.01 | BRCA1 | 12.47 | CYP1B1 | 17.3 |
| HPSE | 7.09 | CAPN3 | 8.36 | NDUFB9 | 10.01 | GNAS | 12.48 | NOX1 | 17.34 |
| RPTOR | 7.09 | RNASE3 | 8.36 | IGF2 | 10.02 | SIGMAR1 | 12.48 | HP | 17.35 |
| MTA1 | 7.1 | PRPH | 8.37 | ACTN2 | 10.04 | IREB2 | 12.5 | LEP | 17.37 |
| CD274 | 7.1 | CFI | 8.37 | ISCU | 10.04 | TGFBR1 | 12.5 | BLVRB | 17.4 |
| ENDOG | 7.1 | LAMP1 | 8.39 | XIAP | 10.05 | CARS2 | 12.51 | NOS1AP | 17.42 |
| BMP4 | 7.1 | BLOC1S1 | 8.39 | CFLAR | 10.05 | CALM3 | 12.52 | FARS2 | 17.5 |
| MTTP | 7.11 | PML | 8.39 | GSTA2 | 10.06 | MAP2 | 12.56 | HRAS | 17.53 |
| TCF7L2 | 7.11 | CCK | 8.39 | NLRP3 | 10.07 | MIF | 12.56 | CALCA | 17.54 |
| TLR6 | 7.11 | TIMP2 | 8.4 | QDPR | 10.08 | CTSD | 12.56 | RAC1 | 17.59 |
| VDR | 7.12 | TRAF2 | 8.41 | CASP2 | 10.1 | MAPKAPK3 | 12.58 | SNAP25 | 17.61 |
| CPQ | 7.12 | STIP1 | 8.42 | SRF | 10.12 | PAH | 12.59 | PRKAA2 | 17.63 |
| NFU1 | 7.12 | NOSTRIN | 8.42 | PKLR | 10.13 | OXR1 | 12.6 | PRKAA1 | 17.66 |
| GADD45G | 7.12 | MYLK | 8.43 | TYMP | 10.14 | CACNB4 | 12.61 | EPO | 17.66 |
| FMO4 | 7.13 | GSTM5 | 8.43 | SCO1 | 10.14 | PXN | 12.61 | MSRB2 | 17.68 |
| BBC3 | 7.14 | TPK1 | 8.43 | PTS | 10.14 | HTR3A | 12.62 | LOX | 17.68 |
| AREG | 7.14 | SLC2A4 | 8.43 | THBS1 | 10.19 | IGF2R | 12.62 | NDUFB8 | 17.72 |
| HK1 | 7.15 | HLA-A | 8.44 | SYP | 10.2 | CASP7 | 12.65 | PDE5A | 17.73 |
| PIK3R2 | 7.15 | UNG | 8.44 | TYK2 | 10.2 | GSTO2 | 12.66 | CRYAB | 17.74 |
| ATG5 | 7.16 | IL3 | 8.46 | GRN | 10.21 | ANG | 12.66 | EGFR | 17.83 |
| POU5F1 | 7.16 | RORA | 8.46 | OPTN | 10.22 | EPHA3 | 12.66 | RPS27A | 17.83 |
| SIRT6 | 7.16 | MDH1 | 8.46 | GRIA1 | 10.22 | PTPN22 | 12.67 | MDM2 | 17.85 |
| MIR214 | 7.17 | RUNX2 | 8.46 | PIK3CB | 10.22 | MRPS16 | 12.67 | THBD | 17.87 |
| JAZF1 | 7.18 | SCARA3 | 8.46 | IKBKB | 10.23 | GLUD2 | 12.74 | NDUFV1 | 17.87 |
| FADD | 7.18 | PDIA3 | 8.47 | CEBPB | 10.23 | CREBBP | 12.75 | CTLA4 | 17.89 |
| BCL2A1 | 7.2 | DDC | 8.47 | CD80 | 10.23 | SLC25A13 | 12.75 | MIR22 | 17.96 |
| IL11 | 7.2 | GSTM4 | 8.47 | C4B | 10.23 | SIRT3 | 12.75 | MAPK9 | 17.98 |
| SELENOT | 7.2 | PIGA | 8.48 | PPP1R15A | 10.23 | GSTA4 | 12.77 | NGF | 18 |
| MAP3K11 | 7.2 | BRCA2 | 8.48 | ACSL4 | 10.24 | IDH2 | 12.79 | IL2 | 18.07 |
| CYP4F2 | 7.21 | UNC13A | 8.49 | EIF2B3 | 10.24 | TOR1A | 12.84 | SRC | 18.08 |
| MIR24-2 | 7.21 | VPS13C | 8.49 | PLA2G4A | 10.25 | DCTN1 | 12.84 | CREB1 | 18.08 |
| ASPA | 7.22 | TNFSF11 | 8.49 | TUBA1B | 10.26 | MIR17 | 12.86 | ATF6 | 18.21 |
| ROCK1 | 7.24 | CYP2C8 | 8.5 | STK11 | 10.27 | C12orf65 | 12.86 | MIR146A | 18.25 |
| NEDD8 | 7.26 | SERPINF1 | 8.5 | MMP3 | 10.27 | EPHX1 | 12.88 | TLR2 | 18.3 |
| GAA | 7.27 | DYSF | 8.51 | GRM5 | 10.29 | TGFBR2 | 12.88 | ATM | 18.34 |
| ATP5PD | 7.27 | PSIP1 | 8.51 | DRD3 | 10.29 | BCHE | 12.91 | SDHD | 18.43 |
| PEPD | 7.28 | TPI1 | 8.52 | SMARCA4 | 10.3 | ERCC6 | 12.93 | IL1A | 18.48 |
| CAPN2 | 7.29 | PDGFRB | 8.52 | NTRK1 | 10.31 | NFE2L1 | 12.93 | NDUFS3 | 18.56 |
| PTPRC | 7.3 | CAMK2G | 8.52 | ECE1 | 10.31 | EIF2AK2 | 12.94 | MT-ATP6 | 18.57 |
| CYP20A1 | 7.3 | EPHX2 | 8.53 | LYN | 10.32 | ADM | 12.95 | MT-CYB | 18.6 |
| MKI67 | 7.31 | CR1 | 8.53 | ELK1 | 10.32 | H2AC18 | 12.96 | CDKN1A | 18.61 |
| SORL1 | 7.31 | CYP3A5 | 8.54 | ALDH3A1 | 10.35 | COL2A1 | 12.98 | OSGIN1 | 18.63 |
| MIR210 | 7.31 | AGRN | 8.54 | PDCD1 | 10.37 | TRPV1 | 12.99 | SLC1A3 | 18.66 |
| SORD | 7.33 | NR2C2 | 8.57 | CCS | 10.38 | VIP | 13 | ATF4 | 18.67 |
| MUC5AC | 7.33 | XRCC6 | 8.58 | SIL1 | 10.39 | SYK | 13 | GTPBP3 | 18.69 |
| ALDH3A2 | 7.33 | PPP3CA | 8.59 | IL5 | 10.4 | A2M | 13.01 | RHOA | 18.71 |
| CHCHD2 | 7.33 | UBQLN1 | 8.59 | PSEN2 | 10.41 | ALOX5 | 13.01 | PPARA | 18.75 |
| PLD1 | 7.34 | FOXO4 | 8.6 | ARG2 | 10.41 | CD44 | 13.02 | ABL1 | 18.77 |
| C5 | 7.34 | MAPK7 | 8.6 | IDO1 | 10.42 | IAPP | 13.02 | KDR | 18.82 |
| MIR222 | 7.35 | NES | 8.6 | DYNLL1 | 10.44 | PRKCA | 13.03 | SPP1 | 18.82 |
| TNFRSF11B | 7.36 | FTL | 8.6 | MTR | 10.45 | SNCAIP | 13.04 | ACAD9 | 18.93 |
| TNFRSF10A | 7.37 | BACH1 | 8.61 | PDIA2 | 10.45 | S100A8 | 13.04 | AGT | 18.99 |
| FABP1 | 7.37 | SCGB1A1 | 8.61 | SLPI | 10.46 | NAGS | 13.04 | TRPM2 | 18.99 |
| PFKM | 7.37 | FMO2 | 8.61 | SLC1A1 | 10.46 | HNRNPA1 | 13.04 | GSTT1 | 19.01 |
| PYCR2 | 7.38 | APC | 8.63 | EIF2B4 | 10.46 | ATP13A2 | 13.05 | PIK3CA | 19.12 |
| IL6R | 7.38 | MYD88 | 8.63 | ADAM17 | 10.48 | SLC25A3 | 13.07 | PRDX1 | 19.19 |
| SUMO1 | 7.39 | HERPUD1 | 8.64 | CAMP | 10.48 | DAPK1 | 13.08 | HMGB1 | 19.19 |
| TEK | 7.4 | CYP17A1 | 8.64 | CYP2A6 | 10.48 | TTR | 13.09 | CLU | 19.2 |
| SLC7A1 | 7.4 | TYRP1 | 8.65 | HDAC1 | 10.48 | EIF4E | 13.11 | PIK3CG | 19.26 |
| FIG4 | 7.4 | XRCC5 | 8.65 | CD79A | 10.52 | DLST | 13.14 | CDKN3 | 19.29 |
| GLT8D1 | 7.4 | FKBP1B | 8.66 | CYP27A1 | 10.52 | CRYAA | 13.14 | HBB | 19.45 |
| LBR | 7.41 | MMP14 | 8.66 | RXRA | 10.54 | UBE2L3 | 13.16 | NDUFA12 | 19.49 |
| CXCL16 | 7.41 | TMEM161A | 8.67 | PTPN1 | 10.55 | NEFH | 13.16 | SELP | 19.5 |
| OSM | 7.42 | ALOX15 | 8.68 | EP300 | 10.57 | G3BP1 | 13.17 | ATP5F1A | 19.57 |
| GRIN1 | 7.42 | SOX2 | 8.68 | CXCR3 | 10.57 | ADH1C | 13.21 | SERPINE1 | 19.6 |
| DYRK1A | 7.42 | ASL | 8.68 | NAT2 | 10.59 | DNMT1 | 13.21 | C1QBP | 19.63 |
| MIR181A2 | 7.42 | CCNF | 8.68 | EDNRA | 10.59 | MRPS22 | 13.23 | PRDX3 | 19.65 |
| DNM2 | 7.43 | DIABLO | 8.69 | PFN1 | 10.59 | TFAM | 13.24 | TXNRD1 | 19.72 |
| TACO1 | 7.43 | MMP7 | 8.69 | MT-ND3 | 10.6 | OSGIN2 | 13.24 | SLC22A5 | 19.73 |
| GIGYF2 | 7.43 | TRPV4 | 8.7 | NTHL1 | 10.6 | CCL3 | 13.25 | MSRB1 | 19.74 |
| C5AR1 | 7.44 | PGK1 | 8.7 | KCNJ2 | 10.61 | SCN4A | 13.26 | AGER | 19.79 |
| ALDH3B1 | 7.44 | NDUFA1 | 8.7 | NRG1 | 10.62 | UCHL1 | 13.26 | PRL | 19.82 |
| CCR7 | 7.44 | IL23A | 8.7 | CHCHD10 | 10.63 | MIR195 | 13.27 | LDLR | 19.85 |
| MUC1 | 7.45 | ATXN1 | 8.71 | BECN1 | 10.64 | FGFR1 | 13.29 | CTNNB1 | 19.87 |
| CANX | 7.45 | JUNB | 8.71 | CLEC4A | 10.66 | OXT | 13.3 | TRDN | 19.88 |
| ADH1A | 7.45 | HSPA14 | 8.71 | COQ2 | 10.66 | MIR34A | 13.32 | CASQ2 | 19.93 |
| FRZB | 7.46 | CD86 | 8.72 | EIF4EBP1 | 10.67 | AKR1A1 | 13.34 | PC | 19.94 |
| EEF2 | 7.47 | GAL | 8.72 | SGK1 | 10.7 | OPA1 | 13.34 | CALM1 | 19.94 |
| ZFAND1 | 7.47 | FGF7 | 8.73 | ANGPT1 | 10.7 | VARS2 | 13.34 | ETFB | 20.01 |
| UBQLN4 | 7.47 | SCP2 | 8.73 | SOCS3 | 10.71 | SLC1A2 | 13.35 | SHC1 | 20.05 |
| PKD1 | 7.47 | BCL6 | 8.73 | UCP1 | 10.71 | CRHR1 | 13.36 | COMT | 20.07 |
| MIR200C | 7.47 | SULT1A3 | 8.74 | MIR93 | 10.72 | POR | 13.37 | ANXA5 | 20.11 |
| TNIP1 | 7.48 | GADD45A | 8.74 | AIF1 | 10.73 | PRKG1 | 13.37 | MMP2 | 20.24 |
| SCN4B | 7.49 | GSN | 8.75 | IL15 | 10.73 | TGFB3 | 13.37 | TH | 20.37 |
| IL16 | 7.5 | MDH2 | 8.75 | TRIM21 | 10.75 | KCNJ5 | 13.37 | SELE | 20.42 |
| H3C14 | 7.5 | IL2RB | 8.76 | LTA | 10.76 | BACE1 | 13.39 | STAT3 | 20.45 |
| ARNT | 7.5 | ABCC8 | 8.76 | NAMPT | 10.77 | SNTA1 | 13.39 | NUDT1 | 20.47 |
| CYP19A1 | 7.51 | PPIG | 8.77 | MIR29A | 10.77 | DRD5 | 13.41 | MT-CO1 | 20.54 |
| SDC1 | 7.51 | MCU | 8.77 | MFN2 | 10.77 | SERPINA3 | 13.41 | MIR21 | 20.55 |
| CACNA2D1 | 7.52 | MLYCD | 8.78 | CALM2 | 10.77 | HSPA9 | 13.41 | EIF2S1 | 20.64 |
| AQP1 | 7.52 | CHMP2B | 8.78 | TIA1 | 10.78 | SCN2A | 13.42 | EIF2AK3 | 20.7 |
| CDC25C | 7.52 | UBC | 8.79 | RELA | 10.79 | AMPD1 | 13.44 | TNFRSF1A | 20.73 |
| ACTN4 | 7.53 | MSN | 8.79 | NGFR | 10.79 | TNFRSF1B | 13.47 | TERT | 20.74 |
| BSG | 7.55 | PTPN3 | 8.79 | TRAP1 | 10.8 | ITPR1 | 13.47 | IL13 | 20.85 |
| LYRM4 | 7.55 | NTF4 | 8.8 | HGF | 10.81 | TSFM | 13.47 | GDNF | 20.89 |
| KL | 7.56 | SLC19A3 | 8.8 | GAP43 | 10.82 | MT-TK | 13.49 | SLC2A1 | 20.92 |
| ANXA11 | 7.56 | CCNB1 | 8.8 | MIR433 | 10.83 | MIR223 | 13.49 | FAS | 20.96 |
| AVP | 7.56 | FMR1 | 8.8 | SLC6A2 | 10.83 | TGFB2 | 13.52 | SOD3 | 21.02 |
| ABCC3 | 7.56 | HSPA6 | 8.81 | CD4 | 10.83 | UCP3 | 13.54 | MB | 21.09 |
| MRAP | 7.56 | HK2 | 8.82 | MYO9A | 10.84 | IDH1 | 13.55 | MT-ND1 | 21.17 |
| FANCD2 | 7.57 | SELL | 8.83 | PENK | 10.86 | CSF1 | 13.55 | SDHA | 21.2 |
| FIS1 | 7.57 | PRKCZ | 8.83 | EPRS1 | 10.86 | DNM1L | 13.55 | CYP1A2 | 21.25 |
| PGAM5 | 7.57 | PTGIS | 8.85 | CDH1 | 10.87 | MET | 13.57 | SDHB | 21.29 |
| CAMKK2 | 7.57 | ZC3H12A | 8.85 | CFTR | 10.88 | TSC2 | 13.63 | NR3C1 | 21.3 |
| CFAP410 | 7.58 | AHR | 8.87 | RPS6KA5 | 10.89 | PLA2G6 | 13.66 | MMP9 | 21.3 |
| VIPR1 | 7.58 | GLS | 8.87 | PVALB | 10.89 | PGD | 13.69 | TXN2 | 21.32 |
| ADRB3 | 7.59 | PPOX | 8.87 | MRPL44 | 10.89 | KCNH2 | 13.69 | OXSR1 | 21.36 |
| RTN4 | 7.59 | NCAM1 | 8.88 | CTSB | 10.91 | HMOX2 | 13.69 | HSPA8 | 21.4 |
| MIR19A | 7.6 | RETN | 8.88 | NTS | 10.91 | PPIF | 13.71 | C9orf72 | 21.45 |
| FAAH | 7.6 | B2M | 8.89 | ERBB4 | 10.92 | EHHADH | 13.73 | GFAP | 21.51 |
| GLA | 7.61 | CD55 | 8.89 | EIF4G1 | 10.93 | TXNRD2 | 13.77 | IGF1 | 21.52 |
| PDE4A | 7.61 | VKORC1L1 | 8.89 | KLF4 | 10.93 | AKR1B1 | 13.78 | TPO | 21.58 |
| TARS2 | 7.61 | SLC25A27 | 8.89 | CYP2B6 | 10.93 | GSTM2 | 13.79 | EGF | 21.61 |
| CX3CR1 | 7.62 | EDNRB | 8.9 | ACE2 | 10.94 | MAP2K3 | 13.79 | MYH7 | 21.72 |
| NOD2 | 7.62 | PF4 | 8.91 | SESN1 | 10.94 | NGB | 13.8 | MSRA | 21.75 |
| SFTPB | 7.62 | SMAD2 | 8.91 | NRF1 | 10.95 | MMP1 | 13.81 | CYP2E1 | 21.76 |
| LANCL1 | 7.63 | SOCS1 | 8.91 | PECAM1 | 10.95 | NCF1 | 13.81 | GCH1 | 21.77 |
| CD38 | 7.63 | BLK | 8.94 | KLF2 | 10.96 | MECP2 | 13.81 | ELAC2 | 21.87 |
| FECH | 7.64 | PYGM | 8.94 | GSS | 10.96 | NPPB | 13.87 | MAOB | 21.96 |
| TPPP3 | 7.65 | CDK6 | 8.95 | EIF2AK1 | 10.96 | SIRT2 | 13.9 | PRDX6 | 22.03 |
| IL1RAPL2 | 7.65 | MICB | 8.95 | FLT1 | 10.96 | STAT1 | 13.92 | CYP1A1 | 22.34 |
| TP53INP1 | 7.65 | PPARD | 8.96 | RPS6KB1 | 10.96 | ECHS1 | 13.93 | PINK1 | 22.38 |
| ENC1 | 7.65 | UBQLN2 | 8.99 | OXA1L | 10.98 | AOC3 | 13.93 | GPX3 | 22.42 |
| PEX5 | 7.66 | HYOU1 | 9 | ATXN3 | 10.99 | H6PD | 13.94 | ACADL | 22.44 |
| IKBKG | 7.66 | TACR1 | 9.01 | HLA-B | 11.01 | FGF2 | 13.94 | SP1 | 22.49 |
| FYN | 7.66 | BDKRB2 | 9.01 | ATF3 | 11.01 | GPX2 | 13.94 | NOX4 | 22.67 |
| ABCG2 | 7.66 | ADRB1 | 9.02 | RAF1 | 11.03 | GLRX2 | 13.95 | CASP9 | 22.79 |
| MIR199A1 | 7.67 | CTTN | 9.03 | MALAT1 | 11.04 | OSER1 | 13.95 | F2 | 22.83 |
| UCN2 | 7.67 | DEPDC5 | 9.03 | ADA | 11.04 | GSTM3 | 13.97 | ETFA | 22.88 |
| CD46 | 7.68 | MIR106B | 9.04 | MAPK11 | 11.04 | KRIT1 | 13.97 | PPARG | 22.88 |
| KIAA0319L | 7.68 | NQO2 | 9.04 | GRIN2B | 11.04 | SDHAF1 | 13.99 | HTRA2 | 22.97 |
| MIR184 | 7.68 | ABCC2 | 9.04 | ANXA2 | 11.06 | OPRM1 | 14.01 | ADIPOQ | 23 |
| CXCL2 | 7.68 | SREBF1 | 9.05 | COX6B1 | 11.08 | SESN2 | 14.05 | CYP3A4 | 23.06 |
| GPX5 | 7.69 | SERPINH1 | 9.05 | HMGCL | 11.08 | APOH | 14.05 | ALDH2 | 23.14 |
| CD28 | 7.7 | H2BC21 | 9.06 | CXCR4 | 11.08 | VDAC1 | 14.06 | FOXO3 | 23.38 |
| YAP1 | 7.7 | LPA | 9.07 | CXCL10 | 11.09 | REN | 14.07 | COX5A | 23.46 |
| GADD45B | 7.7 | VCL | 9.07 | MMP13 | 11.09 | SST | 14.08 | SELENON | 23.56 |
| EIF2AK4 | 7.71 | MIR145 | 9.08 | MIR122 | 11.12 | ADRB2 | 14.09 | OGG1 | 23.62 |
| NEK1 | 7.71 | MIR143 | 9.09 | STK39 | 11.12 | NR3C2 | 14.11 | KNG1 | 23.67 |
| HDAC9 | 7.71 | FASN | 9.1 | GCLM | 11.14 | GSK3B | 14.11 | MTOR | 23.71 |
| ACAD8 | 7.71 | CASP4 | 9.1 | TLR3 | 11.15 | KRAS | 14.15 | CHAT | 23.77 |
| MIR203A | 7.72 | CD34 | 9.11 | BAD | 11.15 | MYC | 14.15 | ABCD1 | 23.89 |
| ADAM10 | 7.72 | KRT18 | 9.11 | TBP | 11.17 | ADH5 | 14.15 | MTO1 | 24.03 |
| MT-CO3 | 7.72 | CTSG | 9.12 | CCN2 | 11.18 | HTR1A | 14.15 | TLR4 | 24.05 |
| RAG2 | 7.73 | SLC11A2 | 9.12 | TJP1 | 11.18 | FXN | 14.16 | BAX | 24.15 |
| C3 | 7.74 | ODC1 | 9.12 | ALDH9A1 | 11.2 | NPPA | 14.16 | PRDX2 | 24.22 |
| HRH2 | 7.75 | TNFRSF10B | 9.13 | BIRC5 | 11.2 | PTK2 | 14.19 | POLG | 24.4 |
| VEGFC | 7.76 | CHEK1 | 9.13 | FCGR3B | 11.21 | CPT1B | 14.19 | MAPK3 | 24.43 |
| SLC8A1 | 7.76 | SFTPD | 9.13 | PDGFRL | 11.21 | HLA-DRA | 14.26 | VCAM1 | 24.45 |
| MRPS34 | 7.76 | CD69 | 9.14 | NLRP1 | 11.22 | UGT1A1 | 14.28 | HSF1 | 24.45 |
| FCGR3A | 7.77 | PDLIM4 | 9.14 | KCNE1 | 11.23 | PRKD1 | 14.3 | NCF2 | 24.62 |
| IL12B | 7.78 | GH1 | 9.14 | GAD1 | 11.23 | CYB5R3 | 14.33 | PRNP | 24.79 |
| CIITA | 7.78 | LPL | 9.14 | ENO2 | 11.24 | GPX4 | 14.34 | FOS | 24.94 |
| SIAH1 | 7.79 | HNF1A | 9.14 | PEX12 | 11.25 | GPT | 14.35 | MAOA | 25.05 |
| MIR23B | 7.79 | SCARB1 | 9.15 | CDKN1B | 11.26 | FMO3 | 14.39 | APOE | 25.05 |
| SET | 7.79 | PTX3 | 9.15 | GJA1 | 11.27 | F3 | 14.39 | HIF1A | 25.17 |
| MIR92A1 | 7.81 | RAD51 | 9.16 | HSPA1B | 11.27 | TFRC | 14.41 | CACNA1C | 25.18 |
| MMD | 7.81 | EZH2 | 9.16 | LOC111365141 | 11.28 | JAK2 | 14.41 | CASP8 | 25.25 |
| ITGA2 | 7.81 | COX15 | 9.16 | FGF1 | 11.3 | MT-TL1 | 14.41 | LMNA | 25.28 |
| TLR5 | 7.81 | LGALS1 | 9.17 | DRD4 | 11.31 | IL1R1 | 14.41 | XBP1 | 25.28 |
| PHYH | 7.82 | GRIN2A | 9.19 | IRF1 | 11.31 | MAP2K1 | 14.45 | CRH | 25.53 |
| HSPG2 | 7.82 | VAPB | 9.2 | ATF2 | 11.31 | MT-CO2 | 14.48 | PPARGC1A | 25.65 |
| PRDM10 | 7.82 | ELAVL1 | 9.2 | CDK1 | 11.33 | ATP2A2 | 14.52 | CAV1 | 26.16 |
| RBP4 | 7.83 | HRH1 | 9.22 | RNF112 | 11.33 | PLA2G7 | 14.52 | PON2 | 26.18 |
| DYNC1H1 | 7.84 | ATR | 9.23 | HMGCR | 11.34 | CD40 | 14.53 | BCL2 | 26.23 |
| HPRT1 | 7.85 | PNKP | 9.24 | DHCR24 | 11.34 | MAP2K4 | 14.56 | TARDBP | 26.28 |
| SPARC | 7.85 | GFER | 9.24 | NTRK2 | 11.34 | GSTO1 | 14.56 | MAPK10 | 26.31 |
| SELENOK | 7.85 | CDH5 | 9.25 | CXCL1 | 11.35 | TPH1 | 14.57 | HSPA1A | 26.33 |
| MAPK8IP1 | 7.86 | MIR24-1 | 9.25 | AHSP | 11.35 | BCL2L1 | 14.58 | ACE | 26.46 |
| TFEB | 7.87 | ABCA1 | 9.25 | NDUFA6 | 11.36 | ACHE | 14.64 | APEX1 | 26.49 |
| TAT | 7.88 | DAXX | 9.25 | PLCG1 | 11.37 | CRAT | 14.66 | OLR1 | 26.49 |
| HDAC2 | 7.88 | CDH2 | 9.26 | IRAK1 | 11.37 | GCLC | 14.68 | ESR1 | 26.5 |
| VNN1 | 7.88 | GZMB | 9.27 | FMO1 | 11.38 | NFKBIA | 14.68 | MAP3K5 | 26.53 |
| MIR142 | 7.88 | CALB2 | 9.28 | HSP90AB1 | 11.39 | SGCB | 14.71 | VCP | 26.6 |
| GDF15 | 7.89 | MUTYH | 9.29 | ITGAL | 11.39 | IL4 | 14.75 | AIFM1 | 26.64 |
| SORCS2 | 7.89 | ERCC8 | 9.29 | AKT2 | 11.4 | UCP2 | 14.77 | ICAM1 | 26.7 |
| MIR152 | 7.89 | DDAH1 | 9.3 | FDXR | 11.41 | NR4A2 | 14.83 | CP | 26.94 |
| SELENOP | 7.89 | NDUFS7 | 9.3 | MIR126 | 11.41 | CD40LG | 14.86 | SCN5A | 26.98 |
| ADSL | 7.9 | NDUFS6 | 9.31 | CCR6 | 11.42 | IL17A | 14.88 | HSPB1 | 27.11 |
| NEFL | 7.9 | ERO1A | 9.32 | KIF1B | 11.43 | EGR1 | 14.89 | BDNF | 27.3 |
| HPX | 7.91 | DSP | 9.32 | DSPP | 11.45 | LOC110806262 | 14.9 | NFKB1 | 27.4 |
| OTC | 7.92 | NDUFA10 | 9.34 | CCNA2 | 11.46 | TRMT10C | 14.92 | PRDX5 | 27.52 |
| LCAT | 7.92 | SUMO2 | 9.34 | CPOX | 11.46 | PNPT1 | 14.92 | HBG2 | 27.57 |
| RYR3 | 7.92 | NRAS | 9.36 | ALDH1A1 | 11.46 | CDKN2A | 14.94 | CAV3 | 27.57 |
| ISG15 | 7.93 | IGF2BP1 | 9.36 | HSPB2 | 11.47 | PTK2B | 14.96 | TYR | 27.6 |
| MAP3K1 | 7.93 | CXCL9 | 9.36 | CNTF | 11.47 | CCL5 | 15.01 | KEAP1 | 27.7 |
| BCR | 7.93 | SLC18A2 | 9.39 | MT3 | 11.48 | NPM1 | 15.05 | GSTM1 | 27.84 |
| SLC25A1 | 7.94 | PIK3C2A | 9.39 | SDHAF2 | 11.5 | ETS1 | 15.05 | AARS2 | 27.95 |
| EPHA4 | 7.94 | BCL2L11 | 9.4 | EPAS1 | 11.5 | PRODH | 15.1 | FOXO1 | 27.96 |
| MAPKAPK5 | 7.95 | STK4 | 9.41 | LONP1 | 11.51 | ACO1 | 15.13 | PSEN1 | 27.99 |
| ACTG1 | 7.95 | HNF4A | 9.42 | ABCC1 | 11.51 | EEF1A1 | 15.17 | DDIT3 | 28.04 |
| CLIC1 | 7.97 | ALOX12 | 9.42 | SETD2 | 11.53 | OGDH | 15.18 | GSTP1 | 28.09 |
| SLC7A11 | 7.98 | GLS2 | 9.43 | E2F1 | 11.55 | TAC1 | 15.23 | CPT1A | 28.1 |
| TLR8 | 7.98 | CFH | 9.46 | MTFMT | 11.59 | CASP1 | 15.27 | SLC25A20 | 28.34 |
| NCF4 | 7.98 | F8 | 9.47 | CALB1 | 11.6 | CYP11B2 | 15.27 | GPX1 | 28.43 |
| DGKQ | 7.99 | DNAJB1 | 9.48 | MBP | 11.61 | NDUFAF2 | 15.28 | SQSTM1 | 28.47 |
| IFNAR1 | 8 | TBK1 | 9.48 | S100A9 | 11.62 | PRKCD | 15.29 | ETFDH | 28.53 |
| MMP8 | 8 | ITIH4 | 9.49 | NDUFS1 | 11.63 | SLC5A7 | 15.29 | HSPA5 | 28.54 |
| CDKN2B | 8 | LRPPRC | 9.49 | IGF1R | 11.66 | SMAD3 | 15.31 | ACOX1 | 28.56 |
| TOP1 | 8 | XRCC1 | 9.5 | CHUK | 11.67 | ENG | 15.32 | CYP2D6 | 28.87 |
| ACO2 | 8.01 | TP73 | 9.5 | DNASE1 | 11.67 | GLUL | 15.33 | VWF | 28.87 |
| MIR25 | 8.02 | ANGPT2 | 9.5 | IFNB1 | 11.7 | HSD17B4 | 15.34 | NDUFS4 | 28.94 |
| CUL3 | 8.03 | AOX1 | 9.51 | VIM | 11.7 | RAC2 | 15.4 | GAPDH | 29.13 |
| DUOX1 | 8.03 | NEAT1 | 9.51 | ANK2 | 11.72 | MT-ND2 | 15.42 | MAPT | 29.16 |
| CAMK4 | 8.04 | NME1 | 9.52 | MAP2K7 | 11.73 | PON3 | 15.42 | PARP1 | 29.43 |
| CUL1 | 8.04 | DES | 9.53 | CR2 | 11.74 | ATXN2 | 15.44 | VEGFA | 29.48 |
| CYB5A | 8.04 | TRPA1 | 9.54 | DLG4 | 11.75 | PTEN | 15.46 | RYR2 | 29.56 |
| IGF2BP2 | 8.04 | TGFA | 9.55 | DRD1 | 11.76 | TSPO | 15.47 | CCL2 | 29.85 |
| UBE2D2 | 8.04 | MAP3K7 | 9.55 | PCNA | 11.77 | ELANE | 15.48 | HADHB | 29.98 |
| FAM120A | 8.05 | EPX | 9.56 | ADCY10 | 11.78 | SDHC | 15.49 | RYR1 | 30.4 |
| AQP4 | 8.05 | PLG | 9.56 | PLAT | 11.79 | TUFM | 15.55 | CYBB | 30.6 |
| PDGFB | 8.05 | INSR | 9.56 | TSC1 | 11.8 | ERN1 | 15.57 | GFM1 | 30.79 |
| MIR27A | 8.05 | RHOD | 9.57 | ELN | 11.81 | POMC | 15.59 | HSPA4 | 31.1 |
| KCNE2 | 8.06 | GP1BA | 9.57 | MBL2 | 11.82 | DBH | 15.61 | PTGS2 | 31.12 |
| HAMP | 8.07 | LGALS3 | 9.58 | BMP2 | 11.83 | CYP2C9 | 15.63 | SNCA | 31.58 |
| BACH2 | 8.08 | CYC1 | 9.58 | LCN2 | 11.85 | GPX7 | 15.63 | IFNG | 31.62 |
| PPP5C | 8.09 | BAG3 | 9.58 | GLUD1 | 11.86 | P4HB | 15.64 | NQO1 | 31.87 |
| AURKA | 8.1 | PARK12 | 9.59 | TNFSF10 | 11.88 | FUS | 15.69 | HSP90AA1 | 31.95 |
| TLR7 | 8.1 | STAT4 | 9.6 | DMD | 11.89 | MT-ND5 | 15.7 | EDN1 | 32.13 |
| PTPA | 8.11 | NR1H4 | 9.6 | MCL1 | 11.89 | FASLG | 15.7 | TGFB1 | 32.32 |
| WRN | 8.11 | DNAH8 | 9.6 | NOL3 | 11.9 | HLA-DRB1 | 15.74 | JUN | 32.45 |
| TTPA | 8.12 | MT-ND6 | 9.6 | CST3 | 11.91 | MIR155 | 15.75 | CYBA | 32.63 |
| RARA | 8.12 | NDUFA9 | 9.61 | CBS | 11.92 | TF | 15.8 | CXCL8 | 32.79 |
| UCN | 8.12 | FCGR2B | 9.61 | NDUFS8 | 11.92 | CHKB | 15.81 | CRP | 33.11 |
| PKP2 | 8.13 | CSK | 9.62 | CHKA | 11.96 | CDK2 | 15.87 | ALB | 33.45 |
| KCNT1 | 8.13 | STK24 | 9.63 | DDAH2 | 11.99 | CD36 | 15.9 | SIRT1 | 33.85 |
| ATXN8OS | 8.13 | AR | 9.63 | NTF3 | 11.99 | HFE | 15.91 | ACADS | 33.91 |
| PARK10 | 8.13 | GYG1 | 9.64 | ACTB | 12 | ACP1 | 15.95 | HADH | 34.03 |
| PARK16 | 8.13 | PRKD2 | 9.69 | PRKAB1 | 12 | GSTA1 | 15.97 | AKT1 | 34.11 |
| PARK21 | 8.13 | PPIA | 9.69 | STUB1 | 12.02 | NOTCH1 | 16.03 | INS | 34.82 |
| MGMT | 8.14 | CDK4 | 9.71 | FCGR2A | 12.02 | DUSP1 | 9.95 | IL10 | 35.03 |
| LIN28B | 8.15 | TPT1 | 9.71 | ITGB2 | 12.04 | PRDX4 | 16.08 | ACADVL | 35.49 |
| SMPD1 | 8.15 | COA8 | 9.72 | MATR3 | 12.05 | PTPN11 | 16.08 | TXN | 35.54 |
| GRM1 | 8.15 | IRF5 | 9.72 | APOB | 12.06 | AGTR1 | 16.12 | CASP3 | 35.93 |
| DMPK | 8.15 | F5 | 9.72 | STK25 | 12.06 | HSP90B1 | 16.13 | G6PD | 36.15 |
| PYCR1 | 8.16 | SCO2 | 9.72 | TLR9 | 12.06 | SLC18A3 | 16.13 | MAPK1 | 36.3 |
| UTRN | 8.16 | PKM | 9.74 | CSF2 | 12.07 | FN1 | 16.15 | SLC6A4 | 36.52 |
| MIR148B | 8.17 | GLO1 | 9.74 | APAF1 | 12.08 | PRKCB | 16.18 | ACADM | 37.37 |
| FOXJ1 | 8.18 | MYH6 | 9.74 | PDYN | 12.08 | CNR1 | 16.19 | IL1B | 37.44 |
| MIR144 | 8.19 | MME | 9.75 | OPRD1 | 12.09 | TREM2 | 16.22 | CYCS | 38.24 |
| CCR5 | 8.2 | FOXM1 | 9.75 | TNFAIP3 | 12.09 | NDUFV2 | 16.23 | MAPK8 | 38.51 |
| SLC40A1 | 8.2 | ACOX2 | 9.76 | ABCB1 | 12.09 | GLRX | 16.24 | HADHA | 38.55 |
| ITGB3 | 8.2 | JAK1 | 9.78 | TGM2 | 12.1 | ITGAM | 16.27 | PRKN | 38.63 |
| CCL11 | 8.21 | H19 | 9.78 | ACTA1 | 12.12 | TECRL | 16.27 | IL6 | 38.95 |
| MECOM | 8.21 | NDRG1 | 9.78 | MIR23A | 12.14 | IL1RN | 16.28 | PON1 | 40.21 |
| MIR20A | 8.21 | PEX11B | 9.79 | GRB2 | 12.17 | CACNA1S | 16.28 | PARK7 | 40.56 |
| DUSP19 | 8.22 | ERBB2 | 9.79 | HSD17B10 | 12.21 | HTT | 16.31 | GSR | 42.22 |
| VASP | 8.22 | FKBP5 | 9.82 | MRPS14 | 12.23 | PLAU | 16.33 | XDH | 43.46 |
| HAO1 | 8.22 | MIR133B | 9.82 | MIR132 | 12.24 | TIMP1 | 16.35 | APP | 43.81 |
| MIR9-1 | 8.22 | TPM1 | 9.82 | LTF | 12.26 | CDK5 | 16.36 | MAPK14 | 44.95 |
| FH | 8.22 | MIR221 | 9.82 | CCL4 | 12.26 | CALR | 16.41 | MPO | 45.08 |
| CYP21A2 | 8.23 | VHL | 9.83 | NDUFS2 | 12.26 | MGST1 | 16.45 | SOD2 | 48.82 |
| CACNA1A | 8.23 | IL2RA | 9.84 | BTD | 12.26 | SUOX | 16.46 | CPT2 | 49.36 |
| BRAF | 8.23 | MSH2 | 9.84 | IFNA1 | 12.26 | PTGS1 | 16.5 | NFE2L2 | 54.46 |
| NOSIP | 8.23 | C4A | 9.86 | RB1 | 12.27 | CYP2C19 | 16.58 | TP53 | 57.5 |
| KLRK1 | 8.23 | RAB5A | 9.86 | SMAD4 | 12.27 | TREX1 | 16.61 | NOS1 | 58.58 |
| ESR2 | 8.24 | PLAUR | 9.86 | MAP2K6 | 12.31 | TTN | 16.61 | HMOX1 | 60.47 |
| PIK3C3 | 8.24 | NEIL1 | 9.87 | SRXN1 | 12.31 | DLD | 16.63 | TNF | 63.9 |
| VTN | 8.24 | SPR | 9.88 | LPO | 12.31 | ADCYAP1 | 16.67 | CAT | 70.77 |
| IL33 | 8.25 | LCK | 9.88 | HBA1 | 12.32 | CS | 16.67 | NOS2 | 71.08 |
| CXCR1 | 8.25 | MSRB3 | 9.89 | IRS1 | 12.32 | TXNIP | 16.67 | SOD1 | 80.41 |
| PRKCG | 8.25 | GLE1 | 9.89 | SLC17A5 | 12.34 | IL18 | 16.67 | NOS3 | 82.97 |
| TALDO1 | 8.25 | TAF15 | 9.91 | KCNQ1 | 12.34 | GCDH | 16.67 |  |  |

**Table S3 Sequences of primers.**

| **Primer name** | **Sequence** |
| --- | --- |
| DUSP1 FP | 5’-TGTAGCACCCCTCTCTACGA-3’ |
| DUSP1 RP | 5’-GACAATTGGCCGAGACGTTG-3’ |
| GAPDH FP | 5’-GCAAGTTCAACGGCACAG-3’ |
| GAPDH RP | 5’-CGCCAGTAGACTCCACGAC-3’ |
| miR-429 FP | 5’- CGCTGCATTAATACTGTCTGGT-3’ |
| miR-429 RP | 5’-CAGTGCAGGGTCCGAGGT-3’ |
| miR-429 RT | 5’-GTCGTATCCAGTGCAGGGTCCGAGGTATTCGCACTGGATACGACACGGCA-3’ |
| U6 FP | 5’-CTCGCTTCGGCAGCACA-3’ |
| U6 RP | 5’-AACGCTTCACGAATTTGCGT-3’ |
| U6 RT | 5’-AACGCTTCACGAATTTGCGT-3’ |

**Table S4 Top 20 KEGG enrichment pathways of the 11 differentially expressed mRNAs.**

| **Term** | **Description** | **Count** | **Q value** | **Genes** |
| --- | --- | --- | --- | --- |
| Hsa05166 | Human T-cell leukemia virus 1 infection | 7 | 1.87x10^-5^ | JUN/ZFP36/NFKBIA/EGR1/TNF/FOS/CDKN1A |
| Hsa05169 | Epstein-Barr virus infection | 7 | 1.49x10^-5^ | VIM/JUN/NFKBIA/TNF/OAS1/TNFAIP3/CDKN1A |
| Hsa05167 | Kaposi sarcoma-associated herpesvirus infection | 8 | 1.22x10^-6^ | JUN/ZFP36/CXCL8/NFKBIA/FOS/CDKN1A/PTGS2/CXCL2 |
| Hsa04621 | NOD-like receptor signaling pathway | 8 | 1.09x10^-6^ | JUN/CXCL8/NFKBIA/TNF/OAS1/TNFAIP3/CXCL2/IL1B |
| Hsa05161 | Hepatitis B | 6 | 4.13x10^-5^ | JUN/CXCL8/NFKBIA/TNF/FOS/CDKN1A |
| Hsa05162 | Measles | 6 | 1.85x10^-5^ | JUN/NFKBIA/OAS1/TNFAIP3/FOS/IL1B |
| Hsa05135 | Yersinia infection | 6 | 1.51x10^-5^ | JUN/CXCL8/NFKBIA/TNF/FOS/IL1B |
| Hsa04380 | Osteoclast differentiation | 6 | 1.49x10^-5^ | JUN/NFKBIA/TNF/FOS/JUNB/IL1B |
| Hsa04668 | TNF signaling pathway | 9 | 1.28x10^-9^ | JUN/NFKBIA/TNF/TNFAIP3/FOS/PTGS2/JUNB/CXCL2/IL1B |
| Hsa04625 | C-type lectin receptor signaling pathway | 5 | 6.08x10^-5^ | JUN/NFKBIA/TNF/PTGS2/IL1B |
| Hsa04620 | Toll-like receptor signaling pathway | 6 | 5.87x10^-6^ | JUN/CXCL8/NFKBIA/TNF/FOS/IL1B |
| Hsa04064 | NF-kappa B signaling pathway | 7 | 6.19x10^-7^ | CXCL8/NFKBIA/TNF/TNFAIP3/PTGS2/CXCL2/IL1B |
| Hsa05146 | Amoebiasis | 5 | 5.82x10^-5^ | CXCL8/TNF/CTSG/CXCL2/IL1B |
| Hsa05142 | Chagas disease (American trypanosomiasis) | 6 | 5.87x10^-6^ | JUN/CXCL8/NFKBIA/TNF/FOS/IL1B |
| Hsa04933 | AGE-RAGE signaling pathway in diabetic complications | 5 | 5.58x10^-5^ | JUN/CXCL8/EGR1/TNF/IL1B |
| Hsa04657 | IL-17 signaling pathway | 9 | 5.13x10^-10^ | JUN/CXCL8/NFKBIA/TNF/TNFAIP3/FOS/PTGS2/CXCL2/IL1B |
| Hsa05323 | Rheumatoid arthritis | 6 | 3.88x10^-6^ | JUN/CXCL8/TNF/FOS/CXCL2/IL1B |
| Hsa05140 | Leishmaniasis | 6 | 1.46x10^-6^ | JUN/NFKBIA/TNF/FOS/PTGS2/IL1B |
| Hsa05133 | Pertussis | 5 | 1.85x10^-5^ | JUN/CXCL8/TNF/FOS/IL1B |
| Hsa05134 | Legionellosis | 5 | 6.14x10^-6^ | CXCL8/NFKBIA/TNF/CXCL2/IL1B |

| **Term** | **Description** | **Count** | **Q value** | **Genes** |
| --- | --- | --- | --- | --- |
| GO:0001228 | DNA-binding transcription activator activity, RNA polymerase II-specific | 6 | 8.00x10^-3^ | JUN/IER2/EGR1/FOS/ATF3/JUNB |
| GO:0048018 | receptor ligand activity | 5 | 3.39x10^-2^ | CXCL8/TNF/OSM/CXCL2/IL1B |
| GO:0005126 | cytokine receptor binding | 5 | 8.00x10^-3^ | CXCL8/TNF/OSM/CXCL2/IL1B |
| GO:0005125 | cytokine activity | 5 | 8.00x10^-3^ | CXCL8/TNF/OSM/CXCL2/IL1B |
| GO:0008138 | protein tyrosine/serine/threonine phosphatase activity | 2 | 3.54x10^-2^ | DUSP1/DUSP2 |
| GO:1990841 | promoter-specific chromatin binding | 2 | 3.54x10^-2^ | ZNF304/EGR1 |
| GO:0016538 | cyclin-dependent protein serine/threonine kinase regulator activity | 2 | 3.54x10^-2^ | CCNL1/CDKN1A |
| GO:0008009 | chemokine activity | 2 | 3.54x10^-2^ | CXCL8/CXCL2 |
| GO:0071889 | 14-3-3 protein binding | 2 | 2.04x10^-2^ | ZFP36/DDIT4 |
| GO:0051019 | mitogen-activated protein kinase binding | 2 | 1.87x10^-2^ | DUSP1/DUSP2 |
| GO:0070412 | rR-SMAD binding | 2 | 1.71x10^-2^ | JUN/FOS |
| GO:0033549 | MAP kinase phosphatase activity | 2 | 8.60x10^-3^ | DUSP1/DUSP2 |
| GO:0017017 | MAP kinase tyrosine/serine/threonine phosphatase activity | 2 | 8.00x10^-3^ | DUSP1/DUSP2 |

**Table S5 GO biological processes of the 11 differentially expressed mRNAs.**

**Table S6 Spearman correlation between immune cell subsets and *DUSP1*.**

| **Gene** | **Immune cells** | **Spearman coefficient** | **P value** | **Type** |
| --- | --- | --- | --- | --- |
| DUSP1 | Neutrophils | 0.338501 | 0.032639 | Positive |
| DUSP1 | T cells CD4 memory resting | 0.269840 | 0.092201 | Positive |
| DUSP1 | Dendritic cells activated | 0.192885 | 0.233077 | Positive |
| DUSP1 | T cells CD8 | 0.162873 | 0.315300 | Positive |
| DUSP1 | Eosinophils | 0.144024 | 0.375275 | Positive |
| DUSP1 | T cells gamma delta | 0.102554 | 0.528889 | Positive |
| DUSP1 | Mast cells activated | 0.068168 | 0.675985 | Positive |
| DUSP1 | B cells naïve | 0.061674 | 0.705390 | Positive |
| DUSP1 | Macrophages M1 | 0.020525 | 0.899963 | Positive |
| DUSP1 | T cells follicular helper | -0.006020 | 0.970591 | Negative |
| DUSP1 | Monocytes | -0.026394 | 0.871570 | Negative |
| DUSP1 | Dendritic cells resting | -0.052216 | 0.748975 | Negative |
| DUSP1 | T cells CD4 memory activated | -0.065195 | 0.689394 | Negative |
| DUSP1 | Macrophages M2 | -0.114767 | 0.480704 | Negative |
| DUSP1 | Macrophages M0 | -0.180150 | 0.265980 | Negative |
| DUSP1 | Plasma cells | -0.202694 | 0.209708 | Negative |
| DUSP1 | NK cells resting | -0.209807 | 0.193815 | Negative |
| DUSP1 | T cells regulatory (Tregs) | -0.210350 | 0.192638 | Negative |
| DUSP1 | T cells CD4 naïve | -0.248882 | 0.121469 | Negative |
| DUSP1 | B cells memory | -0.313792 | 0.048634 | Negative |
| DUSP1 | Mast cells resting | -0.702727 | 0.000000 | Negative |
